# Fitness and transcriptional plasticity of human breast cancer single-cell-derived clones

**DOI:** 10.1016/j.celrep.2025.115699

**Published:** 2025-05-12

**Authors:** Long V. Nguyen, Yaniv Eyal-Lubling, Daniel Guerrero-Romero, Sarah Kronheim, Suet-Feung Chin, Raquel Manzano Garcia, Stephen-John Sammut, Giulia Lerda, Allan J.W. Lui, Helen A. Bardwell, Wendy Greenwood, Hee Jin Shin, Riccardo Masina, Katarzyna Kania, Alejandra Bruna, Elham Esmaeilishirazifard, Emily A. Kolyvas, Samuel Aparicio, Oscar M. Rueda, Carlos Caldas

**Affiliations:** 1 Department of Clinical Biochemistry and Institute of Metabolic Science, School of Clinical Medicine, University of Cambridge, Cambridge, UK; 2 Division of Medical Oncology and Hematology, Princess Margaret Cancer Centre, University Health Network, Toronto, ON, Canada; 3 Department of Medicine, University of Toronto, Toronto, ON, Canada; 4 Department of Medical Biophysics, University of Toronto, Toronto, ON, Canada; 5 Cancer Research UK Cambridge Institute, Cambridge, UK; 6 Breast Cancer Now Toby Robins Research Centre, The Institute of Cancer Research, London, UK; 7 The Royal Marsden Hospital NHS Foundation Trust, London, UK; 8 Centre for Paediatric Oncology Experimental Medicine, Centre for Cancer Evolution: Molecular Pathology Division, The Institute of Cancer Research, Sutton, UK; 9 Department of Molecular Oncology, BC Cancer Research Institute, Vancouver, BC, Canada; 10 Department of Pathology and Laboratory Medicine, University of British Columbia, Vancouver, BC, Canada; 11 Department of Medical Genetics, University of British Columbia, Vancouver, BC, Canada; 12 MRC Biostatistics Unit, University of Cambridge, Cambridge, UK

**Keywords:** breast cancer, clonal heterogeneity, patient-derived tumor xenografts, clonal tracking, cellular barcoding, plasticity, single-cell sequencing, cancer stem cells

## Abstract

Clonal fitness and plasticity drive cancer heterogeneity. We used expressed lentiviral-based cellular barcodes combined with single-cell RNA sequencing to associate single-cell profiles with *in vivo* clonal growth. This generated a significant resource of growth measurements from over 20,000 single-cell-derived clones in 110 xenografts from 26 patient-derived breast cancer xenograft models. 167,375 single-cell RNA profiles were obtained from 5 models and revealed that rare propagating clones display a highly conserved model-specific differentiation program with reproducible regeneration of the entire transcriptomic landscape of the original xenograft. In 2 models of basal breast cancer, propagating clones demonstrated remarkable transcriptional plasticity at single-cell resolution. Dichotomous cell populations with different clonal growth properties, signaling pathways, and metabolic programs were characterized. By directly linking clonal growth with single-cell transcriptomes, these findings provide a profound understanding of clonal fitness and plasticity with implications for cancer biology and therapy.

## Highlights

• In breast cancer PDTX models, rare propagating cancer clones are found
• Propagating cancer clones regenerate the full transcriptional landscape of a PDTX model
• Model-specific differentiation programs drive remarkable clonal transcriptional plasticity
• Cell fractions in basal breast cancer models show distinct fitness and molecular properties

## Graphical abstract

**Figure:**
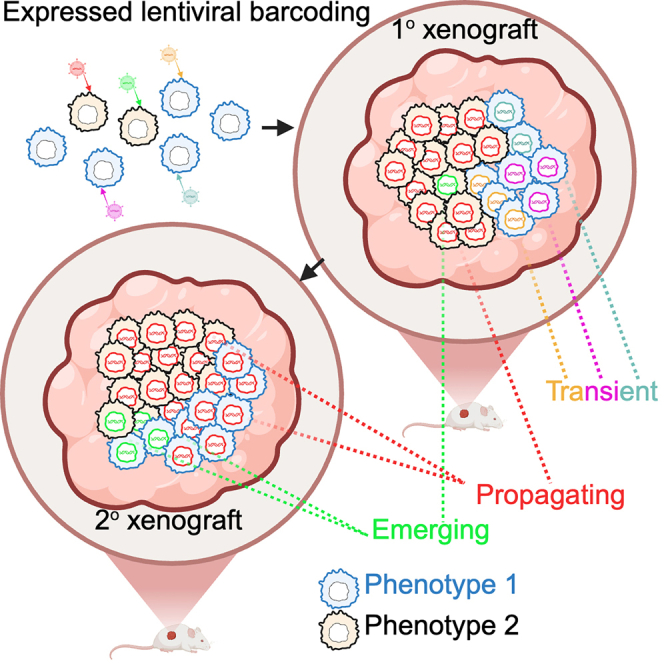

## Teaser

Nguyen et al. used expressed lentiviral-based cellular barcoding coupled with single-cell RNA sequencing to track single-cell-derived cancer clones from patient-derived tumor xenograft (PDTX) models of human breast cancer. Cancer clones demonstrating propagating activity are extremely rare but exhibit remarkable transcriptional plasticity and highly conserved PDTX-model-specific differentiation programs.

## Introduction

Thousands of human cancers have been molecularly profiled, leading to the identification of tumor subtypes with distinct biology and clinical outcomes.^1^^,^^2^^,^^3^^,^^4^^,^^5^ Despite this improved taxonomy, disease progression and treatment resistance remain unsurpassable hurdles for the eradication of many cancers. This is attributed both to the plasticity of pre-existing clones and to the dynamic emergence of subclonal populations.^6^^,^^7^^,^^8^ This has been observed through studies investigating temporal clonal evolution and under selective pressures such as drug treatment.^9^

The concept that not all cells in a tumor can demonstrate clonogenic activity and propagate the cancer is not new.^10^ Perhaps the most robust evidence comes from leukemia, where stem cells that can be isolated based on defined cell surface markers demonstrate both self-renewal and long-term engraftment activity. Arguably, these cells must be eradicated to achieve long-term cures. In solid cancers, attempts have been made to similarly prospectively isolate cells with clonogenic activity,^11^ but there has been a lack of reliable markers to achieve this to the same extent as in hematopoietic malignancies.^12^ As a result, it has been challenging to characterize the functional growth properties of such cells and the molecular mechanisms that regulate their properties.

The introduction of lentiviral-based genetic barcoding strategies has made it feasible to track the progeny from single cells labeled with a permanent DNA-based barcode sequence.^13^ The progeny of these uniquely marked cancer cells can be tracked as single-cell-derived clones through the presence and prevalence of the barcodes detected over time, no longer requiring limiting dilution assays to measure the output from single cells with clonogenic activity. This has allowed for thousands of clones to be tracked simultaneously, revealing significant heterogeneity in clonal growth and propagating activities in both hematologic and solid malignancies.^6^^,^^8^^,^^14^^,^^15^^,^^16^ By combining these cell-tracking approaches with single-cell RNA sequencing (scRNA-seq), where the barcode sequences can be detected from mRNA transcripts, the dynamics of malignant clones can be linked to the transcriptional processes driving their functions.^17^ This has the potential to reveal novel insights into the molecular regulation of clonal growth properties in heterogeneous tumor cell populations. Such an approach has been demonstrated in studies of hematopoietic stem cell regulation,^18^ leukemic stem cells,^19^ and lung cancer progression.^20^^,^^21^

We apply this approach to human breast cancer and present a significant resource of over 20,000 single-cell-derived clones from 26 patient-derived tumor xenograft (PDTX) models tracked with expressible lentiviral barcoding. Our approach is quantitatively rigorous, allowing for both the frequency of clone initiation and the average *in vivo* population doubling times per clone to be calculated across all PDTX models examined. A subset of 17 primary and matched secondary passaged xenografts were further analyzed by scRNA-seq to link clonal growth with transcriptional processes. Propagating clones were found to be extremely rare (<0.01% of cells tested for *in vivo* clonogenic activity), often associated with a fast *in vivo* population doubling time (<10 days between population doublings) and display a highly conserved model-specific differentiation program that is manifested by the reproducible regeneration of the entire transcriptomic landscape of the originating xenograft model. These propagating clones undergo dynamic cell-state transitions revealing remarkable transcriptional plasticity at single-cell resolution. Moreover, basal breast cancer models were found to be composed of dichotomous cell populations that display different clonal growth properties, signaling pathway activation, and metabolic programs, results that have significant implications for our understanding of cancer progression in epithelial malignancies.

## Results

### A resource of 20,000 tracked single-cell-derived clones reveals heterogeneity in clone-initiating activity and rare propagating clones

**Figure 1:**
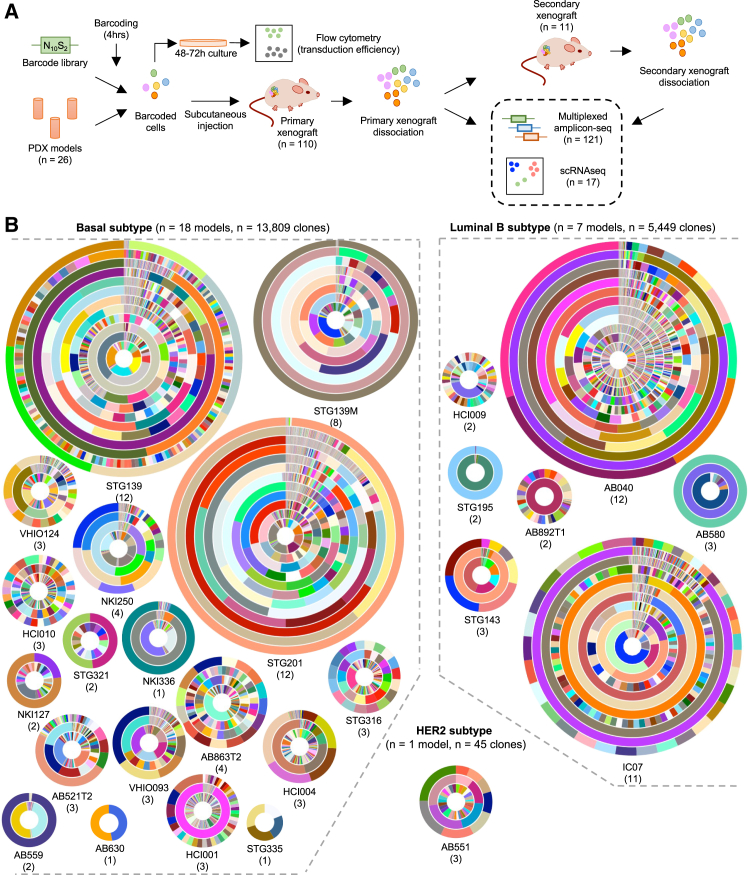
Landscape of 20,000 single-cell-derived clones reveals significant heterogeneity (A) Overall experimental schematic. (B) Overview of all clones detected in primary barcoded xenografts. Each circular diagram represents a single PDTX model, with each concentric circle representing the number and proportion representation of all clones detected in each replicate xenograft in order of highest (innermost circle) to lowest (outermost circle) cell dose implanted. Colors cannot be compared between xenograft model replicates, as none of the clones detected in individual xenografts are related.

We quantified and tracked the cells from human breast cancer PDTX models that can form clones *in vivo*. For this, we applied a lentiviral-based method that genetically labels single cells with a unique, heritable, and expressible DNA-based barcode sequence (Figure S1 and STAR Methods). Notably, for these experiments, a single-cell suspension of PDTX cells was rapidly transduced with the barcode libraries for only 4 h and immediately implanted subcutaneously into NOD.Cg-Prkdc^SCID^ Il2rg^tm1Wjl^/SzJ (NSG) mice to ensure that any *in vivo*-generated clones detected were established from a uniquely barcode-labeled single cell and, further, that it is exceedingly unlikely any cell division occurred within that time span (Figure 1A). Using this approach, we measured the *in vivo* clone-initiating activity from these uniquely barcoded cells in terms of the single-cell-derived progeny they produce, using high-throughput multiplexed targeted amplicon sequencing. Concurrently, the expression profile of the cells constituting these clones can also be analyzed by scRNA-seq, as the barcode sequences are transcribed into mRNA transcripts (Figure 1A).

In total, 110 individual xenografts from 26 PDTX models were analyzed: 18 basal, 7 luminal B, and one HER2 subtype (Figure 1B). Overall, we detected 19,303 clones in primary barcoded xenografts (13,809, 5,449, and 45 in basal, luminal B, and HER2 subtype PDTX models, respectively). We immediately detected significant heterogeneity in clone-initiating cell (CIC) frequency, defined as the number of clones detected in the primary barcoded xenograft as a fraction of the total number of single and uniquely barcoded cells implanted to establish the xenograft. The fast lentiviral transduction method we applied does not allow for growth post transduction, which therefore ensures that every clone detected *in vivo* was derived from a single uniquely barcoded cell. This minimizes potential biases that arise from allowing barcoded cells to expand prior to implantation. CIC frequency was found to be highly variable, ranging from 1 in 53 to 1 in 4,820 cells assayed for luminal B models, and from 1 in 4 to 1 in 3,997 cells assayed for basal models. This variability did not appear to be subtype or hormone-receptor dependent (Table S1) but did appear to be correlated with cell dose. In a linear mixed model, we observed a negative association between CIC frequency and the number of cells implanted (Figure S2A). This negative association appeared similar for the overall dataset of 26 PDTX models, with stronger associations observed in the 5 models for which multiple cell doses were specifically tested (Figure S2B). In these 5 models, the engraftment efficiency was 100% for all models, at all cell doses tested (between 2 × 10^4^ and 1 × 10^6^). A significant likelihood ratio test (*p* = 0.008) implies that the variability in slopes across different PDTX models is most probably biological rather than purely stochastic (STAR Methods). When comparing the rate of tumor growth stratified by cell dose implanted, we observed an increased lag time (i.e., time to first measurable growth) with decreased cell doses, but the exponential growth phases appear to be similar irrespective of cell dose (Figure S3). Furthermore, bulk RNA sequencing showed few differentially expressed genes between xenografts established with high compared to low cell doses, ranging from one to 23 in the majority of PDTX models analyzed (including basal models STG139 and STG201, and luminal B models AB040 and IC07; Figure S4A). The exception to this was basal model STG139M, which showed 87 differentially expressed genes (associated with an increase in oxidative phosphorylation and a decrease in KRAS signaling for xenografts established with high compared to low cell doses; Figure S4B). Overall, we show that cell dose is negatively associated with CIC frequency and, in some cases, can affect the overall expression profile of the xenografts.

The highest CIC frequency of 1 in 4 was calculated from the mean of basal PDTX model STG201 transplanted with 2 × 10^4^ cells per mouse in triplicate. In one of the 3 xenografts analyzed, the CIC frequency was 1 in 1, suggesting that all cancer cells could demonstrate *in vivo* clonal growth activity under these conditions. The same PDTX model when established at a cell dose of 9.2 × 10^5^ cells per mouse yielded a mean CIC frequency of 1 in 2,872 cells (Table S1). Some PDTX models were more sensitive to the effect of cell dose on CIC activity as seen from the different slopes of the negative correlation (Figure S2B). These results further support the finding that CIC activity is, at least in part, cell-dose dependent and thus suggest it can be variable based on non-cell-autonomous factors.

**Figure 2:**
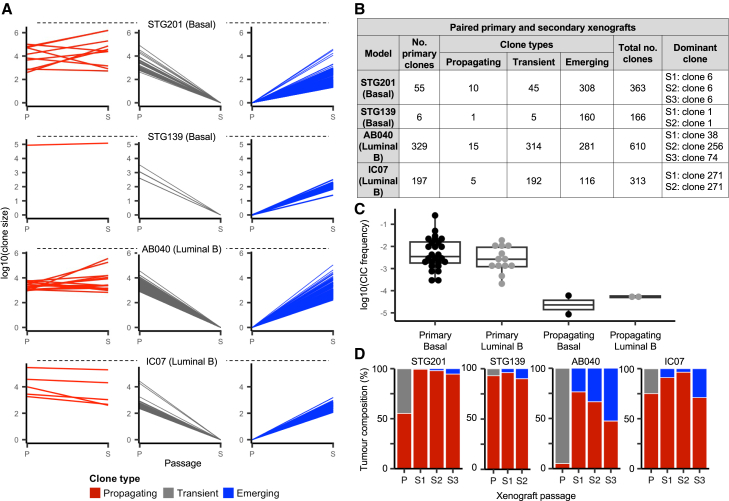
Differences in clone-propagating activity revealed in secondary xenografts (A) Clones detected upon passaging of xenografts. Where a propagating clone was detected in multiple secondary xenograft replicates, the mean clone size is shown. (B) Number of clones detected in primary and secondary xenografts from multiplexed DNA amplicon sequencing. (C) CIC frequency for primary xenografts and frequency of propagating clones are shown for basal (black) and luminal B (gray) models from (A). Boxplots were computed using the median of the observations (center line). The first and third quartiles are shown as boxes, and the whiskers extend to the +/− 1.58 interquartile range divided by the square root of the sample size. Outliers are shown as dots. (D) The proportion of cells represented by propagating, transient, and emerging clones in primary (P) and secondary (S1–S3) xenograft replicates is shown. Same color legend as (A).

To identify the clones that can propagate upon passaging into secondary xenografts, we randomly picked one primary barcoded xenograft from each of four PDTX models (basal models STG139 and STG201, and luminal B models AB040 and IC07) that were established at the highest cell dose in triplicate (Table S1). These were passaged into 2 or 3 secondary replicate mice (S1, S2, and S3). The primary xenografts established with the highest cell doses were chosen because they contained the highest number of clones with the potential to propagate in secondary xenografts. Based on DNA amplicon sequencing, we observed 3 patterns of clonal growth: propagating (i.e., clones detected in both primary and secondary xenografts), transient (i.e., clones only detected in primary xenografts), and emerging (i.e., clones detected in secondary xenografts but not in the primary; Figure 2A). In total, we detected 31 propagating clones, 556 transient clones, and 865 new emerging clones (i.e., below the limit of detection in primary xenografts) (Figure 2B). Strikingly, clones with propagating activity were extremely rare, representing 1 in 115,920 STG139 cells and 1 in 16,560 STG201 cells (both basal models), and 1 in 15,947 AB040 cells and 1 in 22,000 IC07 cells (both luminal B models) (Figure 2C). Propagating clones were frequently dominant (comprising most cells) in the secondary xenograft (Figure 2D). Taken together, it appears that, while, in some circumstances, all cancer cells have CIC activity, the cancer cells with propagating activity are extremely rare and expand to comprise the majority of tumor cells in the secondary xenograft.

### *In vivo* clone doubling time reveals breast cancer subtype-specific differences in clonal fitness

Our experimental approach allows us to quantitatively track the clonal outputs from single uniquely barcoded cells. We therefore computed *in vivo* doubling time for each individual single-cell-derived clone, which considers clone size (to calculate the number of population doublings to reach that size from a single starting cell) and time *in vivo* (to calculate the rate at which these population doublings occur; STAR Methods). Although clone initiation and growth can be affected by the presence of other clones, we devised this metric to compare the growth rate of individual clones independently of variables such as number and size of competing clones within a xenograft (as other clones in a xenograft will alter the proportional representation of the clone of interest). This metric has the advantage of being independent of overall tumor size (as smaller or larger size endpoints would affect clone sizes) and time *in vivo* as well (as shorter or longer times to reach the same clone size would mean inherently different growth rates). We used this metric to compare clonal fitness between clones, xenograft replicates, and PDTX models.

**Figure 3:**
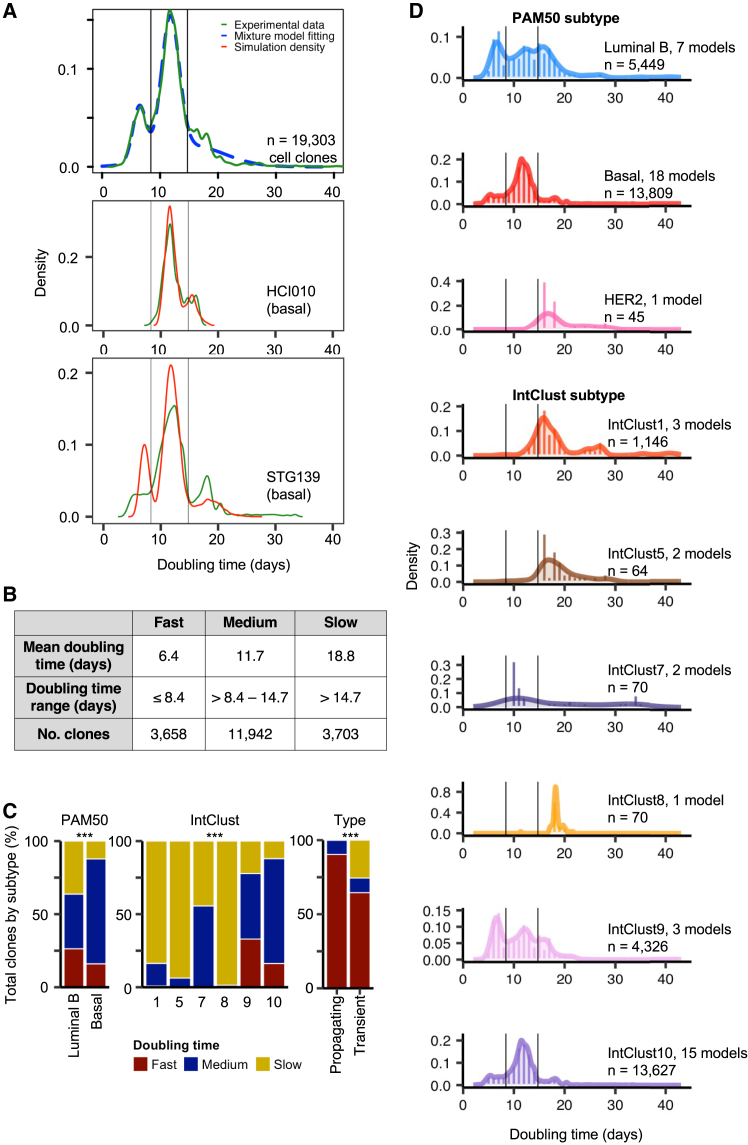
Clone *in vivo* doubling times differ by breast cancer subtype (A) Density distribution of *in vivo* doubling time calculated for all clones across 26 PDTX models (top). *In silico* simulations for models with 2 (middle-HCI010) and 3 (bottom, STG139) distributions are shown. Actual experimental data in green, mclust modeling in blue, and *in silico* simulation in red. (B) Summary of the characteristics of 3 distinct clone types defined by their *in vivo* doubling time. (C) Proportion of all cells from xenografts of each breast cancer subtype that belong to a clone with a fast, medium, or slow doubling time. Asterisks indicate statistical significance from chi-squared test (*p* < 2.2 × 10^−16^), indicating that the proportions of fast, medium, and slow clones are significantly different between subtypes. (D) Distribution of clones based on their *in vivo* doubling times, shown by subtype. Vertical black lines indicate the cutoffs between the Gaussian distributions for fast, medium, and slow clones as defined in (A) and (B).

We analyzed the density distribution of *in vivo* doubling times by merging the data from all clones across all PDTX models and then fitted a mixture Gaussian model to all 19,303 primary clones using the mclust 5 R package^22^ (STAR Methods). This revealed 3 distinct Gaussian distributions (Figure 3A). Employing these distributions, we categorized clones based on their doubling time as fast, medium, or slow (19%, 62%, and 19% of all clones, respectively; Figure 3B). To determine whether this multimodal distribution was the result of biological differences in clone doubling time and not merely stochastic, we performed *in silico* simulation. Starting from single barcoded cells, we modeled cell division per clone over time at different doubling times until reaching 10^7^ total cells, which mimics the maximum permissible tumor size in mice (STAR Methods). This simulation was conducted for each of the 26 PDTX models and demonstrated that *in silico* simulation closely replicated the density distribution of *in vivo* clone doubling times as observed from experimental data (Figures 3A and S5). The results strongly suggest that the observed density distribution is a result of clones that inherently display model-specific *in vivo* doubling times and that such a process is unlikely to be purely stochastic. Moreover, these distributions represent clones with a similar overall *in vivo* growth rate and thus provide a useful framework for classifying the functional growth activity (i.e., clonal fitness) of single-cell-derived clones.

In contrast to CIC frequency, which did not appear to be breast cancer-subtype dependent, clones classified by doubling time exhibited significantly distinctive proportions when analyzed by PAM50 or IntClust subtype (*p* < 2.2 × 10^−16^ and *p* < 2.2 × 10^−16^, respectively; Figures 3C and 3D). In luminal B and basal subtypes, fast- and medium-growing clones were most prevalent (>60% of all clones), in contrast to the HER2 subtype where slow-growing clones were the most prevalent (98% of all clones, although we only have 1 PDTX model representing the HER2 subtype). Analysis by IntClust subtype revealed that IntClust 9 had 78% and IntClust 10 had 88% fast- and medium-growing clones. This contrasts with IntClusts 1, 5, and 8, which predominantly comprised slow-growing clones (84%, 94%, and 99%, respectively). Notably, 26% of clones in the luminal B subtype were fast growing, and almost all of these were confined to IntClust 9 models, despite luminal B models also encompassing IntClusts 1, 5, 7, and 8, where very few or no fast-growing clones were found. We note that IntClust 9 is defined by amplification of *MYC*,^1^ which may explain the predominance of fast-growing clones in these compared to other luminal B models. This suggests that the IntClust subtype stratification is a more accurate predictor of *in vivo* clone doubling time than PAM50 intrinsic subtypes, and that genomic drivers in the form of copy number aberrations as defined by IntClust subtypes can influence clone functional growth properties.

Propagating clones in primary xenografts predominantly had a fast doubling time, 28 out of 31 (90%), which was significantly higher when compared with transient clones, 359 out of 556 (65%) (*p* < 2.2 × 10^−15^; Figure 3C). This suggests that, despite being extremely rare, propagating clones tend to have a fast *in vivo* doubling time and expand to become the majority of cells in the secondary xenograft, further demonstrating their unique functional properties when compared to transient clones.

### Dominant propagating clones regenerate the full model-specific transcriptional landscape

**Figure 4:**
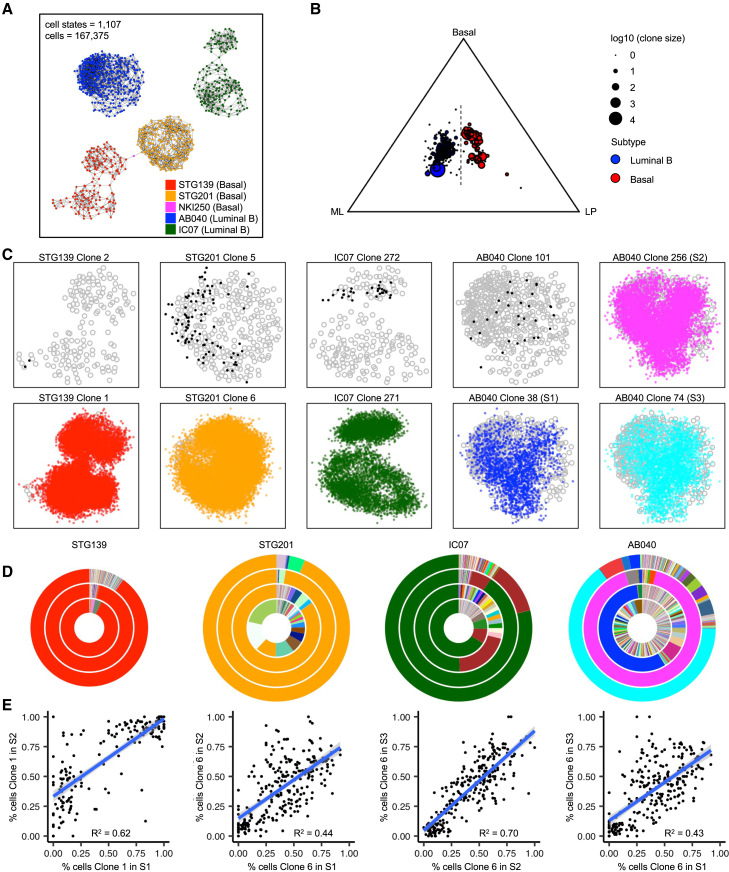
Dominant propagating clones regenerate full PDTX model-specific transcriptional landscape (A) Two-dimensional plot of scRNA-seq data from 5 PDTX models (18 xenografts) showing distinct transcriptional cell states. (B) Ternary plot for each clone detected by scRNA-seq in primary xenografts shows the proportion of total unique molecular indexes (UMIs) for each clone that correspond to normal epithelial gene signatures for basal, LP, and/or ML cells. Vertical dashed line indicates the separation between clones from basal and luminal B models. (C) Distribution of cells per clone by model. Cell states represented by gray open circles. The largest transient clones are in black and the dominant propagating clones are colored by model. AB040 has a different dominant propagating clone in each secondary xenograft replicate, and these are distinctly colored. (D) Number and proportion of all clones detected by DNA amplicon sequencing for each PDTX model. The primary xenograft is represented by the innermost ring, followed by secondary xenograft replicates S1, S2, and S3 in concentric rings outward. The same colors between rings within each PDTX model indicate these are the same clones. The dominant propagating clones are colored the same as (C). (E) Correlation of the proportion contributed to each cell state between secondary xenograft replicates for each dominant propagating clone for STG139 and STG201. Blue lines show the linear correlations, and shaded area indicates the standard error. Adjusted R^2^ is also provided for each correlation.

We obtained 167,375 high-quality single-cell RNA profiles from 5 PDTX models (comprising a total of 17 individual xenografts; 4 of these models with matched primary and secondary passaged xenografts analyzed) (Table S2). In approximately 33% of these, the associated lentiviral barcode was identified, allowing matched clonal growth and transcriptomic analyses (STAR Methods). Single-cell RNA profiles were analyzed using the R package metacell^23^ to group cells with similar transcriptional profiles into distinct metacells, which correspond to different cell states (Figure 4A). The 1,107 cell states identified by this analysis were PDTX-model specific, with no overlap between models, as we and others have previously observed in human tumor scRNA-seq data.^24^^,^^25^ Single cells with or without detected barcodes similarly spanned the spectrum of PDTX transcriptional cell states (i.e., 1,105 out of 1,107 [99.8%] of metacells were represented by barcoded cells; Figures S6 and S7), indicating that the marked clones are representative of the transcriptional heterogeneity in each model. The single-cell expression matrix for all barcoded clones was projected onto gene signatures derived from normal human mammary epithelial cell types^26^: basal, luminal progenitor (LP), and mature luminal (ML; STAR Methods). This approach revealed a separation in ternary plot space based on subtype, largely driven by a higher ML signature contribution for luminal B clones (as opposed to basal clones; Figure 4B). Although cell states were model specific, they demonstrated significant overlap in gene signatures derived from normal human mammary epithelial cell types, revealing that clones from luminal B models could be distinguished from basal models based on a higher ML signature contribution.

We then examined in each PDTX model what was the contribution of each clone to the cell states identified. We observed that across all 4 PDTX models where secondary xenografts had been generated, only the dominant propagating clone (i.e., the propagating clone with the largest contribution of cells in each secondary xenograft) produced cells that spanned all model-specific cell states. Strikingly, no other clones demonstrated the same differentiation capacity, including transient clones, emerging clones, and other non-dominant propagating clones (Figures 4C, S6, and S7). While, in some cases, (clone 1 in basal model STG139 and clone 271 in luminal B model IC07) these clones were already dominant in the primary xenograft, in other cases (clone 6 in basal model STG201 and clone 38 in luminal B model AB040), the clones were not the dominant clone in the primary xenograft. However, in all cases, the propagating clones did not span all cell states in the primary and only demonstrated robust differentiation capacity upon propagation into secondary xenografts (Figure S8). The dominant propagating clones contributed to 100% of metacells containing barcoded cells, whereas the aggregate of cells from all transient clones only contributed to 2 out of 166 (1%), 77 out of 260 (30%), 255 out of 490 (52%), and 20 out of 190 (11%) of metacells for models STG139, STG201, AB040, and IC07, respectively (Figures S6 and S7). This shows that the transient clones have less differentiation capacity, contributing to fewer cell states compared to the dominant propagating clones. Interestingly, the dominant propagating clones consistently demonstrated the highest entropy, calculated using the Shannon index (STAR Methods), followed by the non-dominant propagating clones, then transient clones (Figure S9A). Not surprisingly, the metacells that contained cells from transient clones were found to have a higher entropy value (corresponding to increased diversity of clone representation) (Figure S9B). To complement the metacell representation of these clones, we also generated a uniform manifold approximation and projection (UMAP) for each PDTX model to show the distribution of cells from each clone type across Seurat clusters^27^ (Figure S10). This reinforces the robust differentiation capacity of dominant propagating clones that contribute cells across the full transcriptional landscape of each PDTX model in contrast to non-dominant propagating clones and transient clones, which demonstrate a more limited differentiation capacity.

In three of the models (STG139, STG201, and IC07), the dominant propagating clone was the same across all secondary xenograft replicates (Figure 4D). This suggests these dominant propagating clones harbor an inherent fitness advantage that is reproduced in all secondary xenograft replicates. This was not the case for AB040, where each of the secondary xenograft replicates had a different dominant propagating clone that produced cells spanning all cell states. AB040 differed from the other models because it had the highest number of clones (610 compared to between 166 and 363 for the other models; Figure 2B), and, despite having the most propagating clones, the majority of the primary xenograft was composed of cells from transient clones (95% compared to 5% for propagating clones; Figure 2D). Furthermore, the dominant propagating clones in AB040 (clones 38 and 74) represented only 0.1%–0.4% of cells in the primary xenograft compared to 93%, 10%, and 64% for the dominant propagating clones in models STG139, STG201, and IC07, respectively (clones 1, 6, and 271).These results suggest that multiple clones present in the initial primary xenograft for AB040 had the inherent capacity for robust differentiation, and which clone would go on to demonstrate this capacity in secondary xenografts was a more stochastic process, where only one dominant propagating clone would fulfill this role at a time.

Intriguingly, the fraction of cells in each cell state was highly similar across secondary xenograft replicates for the dominant propagating clones from STG201 and STG139 (Figures 4E and S8), although this was much less so for AB040 and IC07 (Figures S8 and S11). This suggests there is a conserved differentiation program manifested by dominant propagating clones across secondary xenograft replicates, particularly in basal PDTX models.

Altogether, these results demonstrate that dominant propagating clones have an inherent fitness advantage and a robust differentiation capacity to produce cells spanning the full transcriptional spectrum of cell states.

### Dichotomous cell populations in basal breast cancer distinguish between functional clone types based on differential signaling and metabolic responses

**Figure 5:**
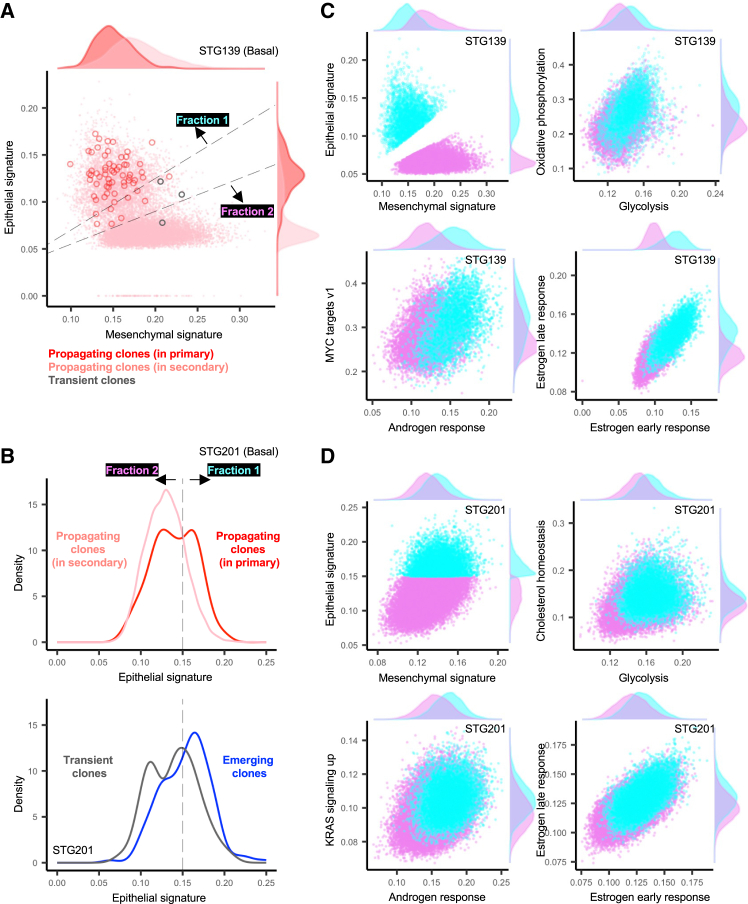
Dichotomous cell populations with differential signaling and metabolic responses in basal breast cancer distinguish between functional clone types (A) Epithelial and mesenchymal signature expression (from scRNA-seq) for all barcoded cells in STG139 color coded by clone type. Dashed lines indicate the threshold above and below which cells belonging to fraction 1 and fraction 2 are defined. Histogram density plots are also shown. (B) Density distribution of all barcoded cells from STG201 colored by clone type. The vertical dashed line defines the threshold above and below which cells belonging to fraction 1 and fraction 2 are defined, based on an epithelial signature score of 0.15, coinciding with the inflection point between the bimodal distribution in epithelial signature score for propagating clones analyzed in the primary xenograft. (C) Scatterplots compare signature expression levels for fraction 1 (cyan) and 2 (magenta) from STG139 related to (A). Density plots are also shown. (D) Same as (C) but for STG201.

To investigate whether gene expression can distinguish between propagating and transient clones, we used the MSigDB Hallmark gene sets to calculate gene signature scores for every cell and compared these single-cell gene signature scores annotated by clone function. These analyses revealed both basal models, STG139 and STG201, had dichotomous cell populations. In STG139, the two distinct phenotypic cell populations were characterized by high epithelial/low mesenchymal and low epithelial/high mesenchymal signatures (Figure 5A). Strikingly, cells from propagating clones in the primary xenograft had high epithelial/low mesenchymal signature, and, upon propagation in secondary xenografts, the progeny of these clones was formed of both phenotypic cell populations. In STG201, cells from propagating clones in the primary xenograft demonstrated a bimodal density distribution based on epithelial signature expression. The population of cells with a lower epithelial signature overlaps with the majority of cells from transient clones, whereas the cells with a higher epithelial signature correspond to the majority of cells from emerging clones (Figure 5B). This suggests that cells with a high epithelial signature are associated with actively expanding clones, and may be the more primitive cell population, which is consistent with the high epithelial/low mesenchymal cell population identified in model STG139 corresponding to propagating clones that give rise to progeny of both cell populations.

We further investigated the differences between these dichotomous cell populations in STG139 and STG201 by dividing them into fractions 1 and 2, corresponding to a high epithelial and low epithelial signature score, respectively (Figures 5A and 5B). This analysis revealed hundreds of differentially expressed genes, and, as expected, epithelial mesenchymal transition was the top differentially expressed gene set in both models (Figure S12; Tables S3 and S4). Fraction 1 cells demonstrated significantly higher levels of signaling from MYC targets v1 gene signature in STG139, significantly higher levels of KRAS signaling in STG201, and significantly higher levels of androgen and estrogen-response signaling in both models (Figures 5C and 5D, *p* < 2.2 × 10^−16^ for all comparisons). Fraction 1 cells also demonstrated significantly higher levels of oxidative phosphorylation in STG139, cholesterol homeostasis in STG201, and glycolysis in both models, consistent with a more active signaling and metabolic state (Figures 5C and 5D, *p* < 2.2 × 10^−16^ for all comparisons).

To our knowledge, there are no gene signatures that can be used to predict clone-propagating activity. To identify such a gene signature, we performed differential gene-expression analysis in primary xenografts (STAR Methods) and restricted this analysis to metacells unique to propagating and transient clones. This was performed for models STG139, STG201, and AB040 (IC07 did not have metacells unique to propagating and transient clones; Figure S13; Table S5. Differentially expressed genes comparing cells from propagating and transient clones in basal model STG139, Table S6. Differentially expressed genes comparing cells from propagating and transient clones in basal model STG201, Table S7. Differentially expressed genes comparing cells from propagating and transient clones in luminal B model AB040). Interestingly, we observed 5 gene sets enriched in propagating compared to transient clones in all models analyzed. These gene sets were associated with cell cycle: E2F targets, G2M checkpoint, mitotic spindle, apoptosis, and p53 pathway (Figure S14). In the luminal B model AB040, we observed gene set enrichment for MYC targets v1, IL2 STAT5 signaling, and DNA repair, whereas, unique to the two basal models, we observed gene set enrichment for epithelial-to-mesenchymal transition, TNFA signaling via nuclear factor κB (NF-κB), androgen response, inflammatory response, oxidative phosphorylation, and KRAS signaling. This was consistent with the gene-expression differences observed between the dichotomous cell populations (fractions 1 and 2) observed in basal models. Ultimately, signaling pathways that are differentially expressed between propagating and transient clones appear to be either breast cancer-subtype specific or model specific.

In summary, these results demonstrate that dichotomous cell populations co-exist in basal breast cancer models, these are associated with different functional clonal growth properties that may be regulated through signaling pathway activation and metabolic programs, and there can be a conversion from one phenotype to another upon clone propagation.

### Dynamic transcriptional plasticity of dominant propagating clones

The nature of our dataset is that all cells within the clones observed are the progeny of a single cell and result from a limited and relatively small number of cell divisions. Furthermore, the cell progeny can be temporally ordered based on whether cells are isolated from the primary or secondary xenografts. The analyses of single-cell RNA profiles within these progenies enables characterizing the dynamics of transcriptional plasticity in a single-cell-derived clone using a transcriptional similarity framework to analyze the dynamics of transcriptional processes within clones as they evolve. We show this analysis for dominant propagating clones from PDTX models STG139 and STG201 where the change in expression of defined gene modules (GMs) across transcriptional distances was examined. These GMs are defined based on non-hierarchical clustering of strong and highly variable genes per model (STAR Methods).

**Figure 6:**
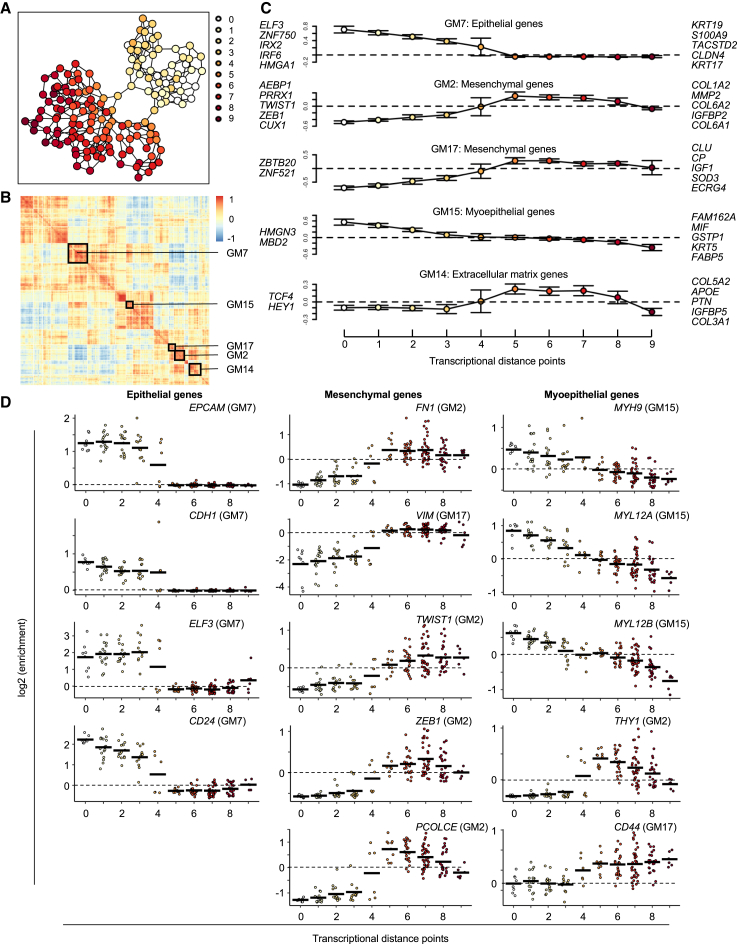
Transcriptional similarity analysis of a dominant propagating clone reveals dynamic transcriptional plasticity (A) Transcriptional cell states represented in a two-dimensional plot colored by transcriptional proximity. (B) Gene-gene correlation plot where strong and highly variable expressed genes are clustered into 18 gene modules (GMs). (C) Fold-enrichment (*y* axis) over transcriptional distance. Error bars are standard error of mean. Dotted horizontal line indicates no enrichment. Top 5 enriched transcription factors and genes within the GM are indicated on the left and right of the plots, respectively. (D) Gene enrichment plots over transcriptional distance. Each data point represents a single cell state. Horizontal black bars indicate the mean log2 enrichment. Horizontal dashed line indicates no enrichment. All plots shown are for clone 1 from basal model STG139.

In the dominant propagating clone for STG139, a total of 18 GMs was identified (Figures 6A and 6B). GM7 and GM2 are of particular interest because they correspond to epithelial and mesenchymal genes, respectively. In GM7, *ELF3* is the top enriched transcription factor, and *KRT19*, *CLDN4*, and *KRT17* are among the top 5 enriched genes (Figure 6C). Other important epithelial genes in GM7 include *EPCAM*, *CDH1*, and *CD24*, which show a gradual decrease in enrichment over transcriptional distance (Figure 6D). In GM2, *PRRX1*, *TWIST1*, and *ZEB1* are among the top 5 enriched transcription factors, and *MMP2*, *IGFBP2*, and collagens *COL1A2*, *COL6A1*, and *COL6A2* are the top 5 enriched genes. These and other important mesenchymal genes, including *FN1*, *VIM*, and *PCOLCE*, show a gradual increase in enrichment over transcriptional distance (Figure 6D). Myoepithelial genes in GM15 (*MYH9*, *MYL12A*, and *MYL12B*), along with several keratin and claudin genes, also show a gradual decrease in enrichment over transcriptional distance, corresponding with a gradual increase in enrichment for a number of collagen genes, matrix metalloproteinase genes (*MMP2*, *MMP11*, and *MMP14*), and mesenchymal genes (*VIM* and *PCOLCE2* in GM14) in this same dominant propagating clone (Figures 6D, S15, and S16). Altogether, this pattern indicates an initial decrease in epithelial and myoepithelial genes coinciding with a gradual increase in mesenchymal gene expression, suggesting that this dominant propagating clone from STG139 undergoes an epithelial-to-mesenchymal cell-state transition upon growing in secondary xenografts.

We identified 11 GMs for the dominant propagating clone from STG201 with a different pattern of transcriptional plasticity. This dominant propagating clone demonstrated a gradual increase in genes associated with an epithelial phenotype, such as *ELF3*, *MUC16*, and several keratin genes in GM7. There was also a gradual increase in genes associated with a mesenchymal phenotype such as *FN1* and collagen genes in GM6 (Figures S17 and S18).

These results illustrate the dynamic nature of transcriptional plasticity in propagating clones, highlighting their ability to evolve, adapt, and mature into dominant clones that constitute the majority of tumor cells in secondary xenografts, all originating from a single barcoded cell.

## Discussion

The cancer clonal and transcriptome resource we present here is valuable in its breadth and rigorous quantitative data representing 20,168 single-cell-derived clones cumulatively tracked for over 200 million individual cell divisions and coupled with 167,375 high-quality single-cell transcriptomic profiles across 26 distinct PDTX models. By ensuring that each clone is derived from a single uniquely barcoded cell, we can characterize the progeny produced from individual cells and, in so doing, showcase the significant heterogeneity that exists in single-cell outputs within PDTX human breast cancer models and derive insight into the transcriptional programs important for regulating the clonogenic activities observed. The interpretability of our dataset is distinguished from others that permit *in vitro* expansion prior to engraftment,^8^^,^^19^ in which case the barcode outputs measured are an average of the expanded cell populations that carry the same barcode. In this latter approach, genomic drivers are more likely to be the cause for differences observed in growth activity of barcode clones and make it difficult to appreciate the effect on clonal outputs by dynamic transcriptional cell states.

A particularly striking finding reported herein is the remarkable ability of a single cell to produce progeny that, within a few cell generations, spans a very broad transcriptional landscape, at times representing all the cell states present within the originating xenograft model. This activity is demonstrated only by dominant propagating clones with a fast *in vivo* doubling time, only one dominant propagating clone at a time, and their differentiation capacity was observed to be remarkably conserved between secondary xenograft replicates. This suggests that dominant propagating clones have an inherent fitness advantage and conserved differentiation program that further distinguishes them from the abundance of transient, emerging, and non-dominant propagating clones that do not demonstrate similar growth properties or transcriptional plasticity.

We observe that, under some conditions, all cells from a basal model of breast cancer can initiate clones *in vivo*, similar to the high clonogenic frequencies observed in a study of melanoma using similarly immunodeficient NSG mice.^28^ However, the majority of these clones in breast cancer models have very limited proliferative activity and are likely to be inconsequential for disease progression. We show that clones with the ability to propagate the cancer from one xenograft to another were extremely rare, between 1 in 115,920 and 1 in 15,947 cells, meaning that less than 0.01% of barcoded cells assayed demonstrated propagating activity. How these frequencies translate to disease *in situ* in patients is yet to be determined, as experimental platforms to study these properties have traditionally relied on severely immunodeficient mice, the most permissive environment for clonal growth from xenografts. The use of a xenotransplant model where cells are engrafted into a foreign microenvironment may limit the ability of clones to propagate. As such, studies using a similar approach in syngeneic mouse cancer models will overcome this limitation. Furthermore, our models have all been propagated subcutaneously, rather than in an orthotopic site. To allay concerns that the site of implantation affects clonal growth, we have previously demonstrated that these models retain the original histological and gene-expression profiles of the originating breast cancers^29^ and that intra-tumor genomic clonal architecture is maintained even when examined at single-cell resolution.^30^

One concept that our findings shed light on is that clonogenic activity *in vivo* can oscillate between active and dormant states. The observed patterns of clonal propagation (propagating, transient, and emerging) align with findings from other studies,^6^^,^^15^ where emerging clones convert from an initially dormant state to an active state. While the definition of emerging clones is dependent on the limit of detection, by definition, these clones must be present in the primary xenograft (but below the limit of detection) and then emerge and form larger more detectable clones in the secondary xenograft. We further provide evidence that this oscillation between active and dormant states is highly influenced by non-cell-autonomous factors, such as the number of co-implanted cells. This is seen as a negative association between cells implanted and CIC frequency (which is also consistent with a bottleneck effect of clone initiation in primary xenografts). However, when there is a greater degree of suppression (i.e., a lower CIC frequency), more emerging clones appear upon secondary passage. This suggests that, despite the suppression of clone initiation, these cells do not expire but can remain in a dormant state until an environment more conducive to clonal growth appears (i.e., in secondary xenografts). This concept of cell-state transitions has been explored before in terms of stochastic cell-state transitions to maintain a phenotypic equilibrium in human breast cancer cell lines.^31^ Here, we expand on this further by demonstrating that dichotomous phenotypic cell populations co-exist in basal PDTX models of human breast cancer, and that they are associated with different functional clonal growth properties. The cell population defined by a high epithelial gene signature score is likely to represent the more primitive cell fraction that is enriched for propagating and emerging clones and gives rise to all other cells. This is consistent with the transcriptional similarity analysis showing dynamic transcriptional plasticity within these dominant single-cell-derived clones, suggesting that there can be a conversion from one phenotype to another upon clone propagation. This primitive cell population was found to be more metabolically active and to have increased MYC and KRAS signaling and increased androgen and estrogen-response signatures. It is further interesting that propagating clones from basal breast cancers that are not typically thought to respond to anti-estrogen therapies were found to have increased estrogen-response signatures compared to transient clones. As these phenotypic differences were detected by scRNA-seq, it is yet to be seen whether these cell populations can be prospectively isolated by cell-separation methods. In some cases, the distinction in gene signatures, although statistically significant, is quite small and therefore likely to only be appreciated by the resolution enabled by single-cell transcriptomic profiling. This further exemplifies how a combined clonal tracking and single-cell transcriptomic profiling approach has revealed profound insights into the molecular regulation of clone function that were not possible to decipher through less sensitive approaches.

The highly dynamic cell-state transitions on a clonal level we observe are also reminiscent of other epithelial cancers. In melanoma, cells with an epithelial phenotype have been implicated in primary tumor growth, while cells with a mesenchymal phenotype constitute a pool of metastatic initiating cells that switch to an epithelial phenotype upon forming and growing metastases.^32^ In a similar way, inhibition of epithelial-to-mesenchymal transition in skin squamous cell carcinoma decreased the incidence of metastasis.^33^ In pancreatic cancer, intermediate states during epithelial-to-mesenchymal transition revealed increased plasticity.^34^ These studies are consistent with the notion that tumors with a mesenchymal phenotype are more aggressive than epithelial tumors, although we have not examined whether cells with a mesenchymal phenotype are more predisposed to metastasize in our PDTX models. Together, these findings and our data suggest that epithelial-to-mesenchymal transition can play a major role in cancer cell plasticity in relation to initiation, progression, and therapy resistance.^35^

The growing focus on molecular-profiling technologies, including single-cell analyses in oncology, aims to refine precision-medicine approaches to cancer treatment.^36^ Our results showing that clones can demonstrate significant transcriptional and functional plasticity warrant further investigation into how this plasticity is modulated by treatment and the potential it holds for unveiling novel strategies to eradicate the cell states most implicated in treatment failure. Future treatment strategies can, for instance, be designed to target signaling and metabolic pathways observed to be upregulated in clones responsible for cancer propagation. Our approach can be applied to study other solid cancers and sets the stage for the development of novel therapeutic strategies that can target and attenuate clonal growth and clonal propagation.

### Limitations of the study

In this study, we aimed to keep the lentiviral transduction efficiency to less than 30% to minimize the number of cells with multiple barcode integrations. This is a technical limitation that restricts the number of clones that can be tracked in any individual xenograft. In one of the models, STG139M, the transduction efficiency was 56%, which can affect the clone number and frequency calculations due to an increased incidence of multiple barcode integrations. The breast cancer subtypes represented by the PDTX models in this study are also skewed toward basal and luminal B breast cancers because these patient tumors have the highest engraftment efficiency in mice. We include 1 HER2 PDTX model and no luminal A models, which limits our ability to make conclusions about their biology. Lastly, while we demonstrate the remarkable differentiation capacity and dynamic transcriptional plasticity of rare propagating clones, our study does not include functional experiments. This is an area for future research that will shed light on the molecular pathways that are required for clone-propagating activity.

## Resource availability

### Lead contact

Requests for further information and resources should be directed to and will be fulfilled by the lead contact, Long V. Nguyen (long.nguyen@uhn.ca).

### Materials availability

This study did not generate new PDTX models. All unique reagents generated in this study are available from the lead contact with a completed materials transfer agreement.

### Data and code availability

• Data generated in this study, including processed count matrices from both bulk and scRNA-seq, can be accessed through Zenodo (https://doi.org/10.5281/zenodo.10978989) and are publicly available as of the date of publication.
• Code to reproduce the mathematical modeling and scRNA-seq analysis is available at https://github.com/cclab-brca/clone-dynamics and is publicly available as of the date of publication.
• Any additional information required to reanalyze the data reported in this paper is available from the lead contact upon request.

## Acknowledgments

L.V.N. is a Hold’em for Life Early Career Professor in Cancer Research in the Temerty Faculty of Medicine, University of Toronto, and was supported by an ESMO Translational Research Fellowship, an ASCO Conquer Cancer Young Investigator Award, a J.P. Bickell Foundation Medical Research grant, a MOHCCN Clinician-Scientist Award, and the Allan Slaight Breakthrough Fund at the 10.13039/100009812Princess Margaret Cancer Foundation. O.M.R. was supported by the 10.13039/100014013UKRI grant MC_UU_0002/16. C.C. was supported by funding from 10.13039/501100000289CRUK (grant numbers A17197, A27657, and A29580), an NIHR Senior Investigator Award (grant number NF-SI-0515-10090), and a European Research Council Advanced Award (grant number 694620). D.G.-R. is funded by the Cambridge Commonwealth, European and International Trust. We are grateful for the generosity of all the patients who donated samples for the development of the tumor xenograft models and the CRUK Cambridge Institute Core Facilities (Genomics, Flow Cytometry, Histopathology, and Biorepository) for support during the execution of this project. The authors dedicate this publication in the memory of Dr. Connie J. Eaves, a phenomenal mentor and a pioneer in the field of cancer stem cell biology.

## Author contributions

L.V.N. and C.C. conceived the study, led data analysis, and wrote the manuscript. Tumor xenograft experiments were designed and led by L.V.N. with input from S.-F.C., A.J.W.L., G.L., H.A.B., W.G., H.J.S., A.B., and E.E. Tumor processing for sequencing was led by L.V.N. with input and expertise contributed by S.-F.C., H.A.B., and K.K. Computational analysis was led by Y.E.-L. and L.V.N. with input from S.K., R.M.G., S.-J.S., and R.M. Mathematical modeling was performed by D.G.-R. with expertise provided by O.M.R. and S.A. All authors read and approved the manuscript.

## Declaration of interests

C.C. was in the past a recipient of research grants (administered by the University of Cambridge) from Genentech, Roche, AstraZeneca, and Servier.

## STAR★Methods

### Key resources table

**Table:** 

REAGENT or RESOURCE	SOURCE	IDENTIFIER
**Bacterial and virus strains**		

pLARRY-EGFP plasmid	Rodriguez-Fraticelli et al.^18^ and Weinreb et al.^37^	https://www.addgene.org/140025/
psPAX2 plasmid	Didier Trono lab	RRID:Addgene_12260
pMD2.G plasmid	Didier Trono lab	RRID:Addgene_12259

**Biological samples**		

PDTX models	Bruna et al.^29^	See Table S1

**Chemicals, peptides, and recombinant proteins**		

Lipofectamine 3000 Reagent	Thermofisher	Cat #L3000001
Q5 high-fidelity DNA polymerase	New England Biolabs	Cat #M0491S
Lenti-X concentrator	Takara Bio	Cat # 631232
Matrigel, Growth Factor Reduced	Corning	Cat # 354230

**Critical commercial assays**		

Tumor Dissociation Kit, Human	Miltenyi Biotec	Cat # 130-095-929
Mouse Cell Depletion Kit	Miltenyi Biotec	Cat # 130-104-694
prepGEM Universal DNA extraction kit	ForenteQ Limited	Cat # PUN0500
IDT for Illumina UD Indexes	Illumina	Cat # 20091654
KAPA Library Quantification Kit	Roche	Cat # 07960140001/KK4824
TruSeq stranded mRNA library preparation kit	Illumina	Cat # 20020594

**Deposited data**		

Bulk RNA sequencing raw count matrices	https://doi.org/10.5281/zenodo.10978989	RawCounts.csv
Bulk RNA sequencing normalized count matrices	https://doi.org/10.5281/zenodo.10978989	LogCPMNormCounts.csv
scRNAseq metacell processed count matrix	https://doi.org/10.5281/zenodo.10978989	mat.pdx_LN_v2_filt.Rda
scRNAseq metacell partitions	https://doi.org/10.5281/zenodo.10978989	mc.pdx_LN_v2_filt.Rda mc2d.pdx_LN_v2_filt.Rda
scRNAseq Seurat processed count matrices	https://doi.org/10.5281/zenodo.10978989	STG139.rds STG201.rds AB040.rds IC07.rds

**Experimental models: Cell lines**		

HEK293T cells	ATCC	CRL-11268
MDA-MB-231 cells	ATCC	HTB-26

**Experimental models: Organisms/strains**		

NOD.Cg-Prkdc^SCID^ Il2rg^tm1Wjl^/SzJ mice	Charles River	RRID:BCBC_4142

**Oligonucleotides**		

Pair 1 forward primer: TCGTCGGCAGCGTC AGATGTGTATAAGAGACAGTAGAAGGCAC AGGTCGACAG	Integrated DNA technologies	N/A
Pair 1 reverse primer: GTCTCGTGGGCTCG GAGATGTGTATAAGAGACAGGTCTAGACT CACTGGCCGTC	Integrated DNA technologies	N/A
Pair 2 forward primer: TCGTCGGCAGCGT CAGATGTGTATAAGAGACAGGCAACTAG AAGGCACAGGTC	Integrated DNA technologies	N/A
Pair 2 reverse primer: GTCTCGTGGGCTC GGAGATGTGTATAAGAGACAGGACTCAC TGGCCGTCGTTTT	Integrated DNA technologies	N/A
Pair 3 forward primer: TCGTCGGCAGCGT CAGATGTGTATAAGAGACAGCAACTAGA AGGCACAGGTCG	Integrated DNA technologies	N/A
Pair 3 reverse primer: GTCTCGTGGGCTC GGAGATGTGTATAAGAGACAGAGACTCA CTGGCCGTCGTTT	Integrated DNA technologies	N/A

**Software and algorithms**		

Metacell version 0.3.7	Baran et al.^23^	https://tanaylab.github.io/metacell/
Seurat version 5.1.0	Satija et al.^27^	https://satijalab.org/seurat/
Custom code	This paper	https://github.com/cclab-brca/clone-dynamics
RStudio version 2023.12.0 + 369	Posit Software, PBC	https://posit.co/download/rstudio-desktop/
FastQC version 0.11.9	Andrews, S.^38^	N/A
Cutadapt version 1.10	Martin, M.^39^	N/A
edgeR version 3.32.1	Chen et al.^40^	N/A
UCell version 2.2	Andreatta and Carmona.^41^	N/A
Pathfinder version 2.3.0.9000	Ulgen et al.^42^	N/A
Mclust version 6.1.1	Scrucca et al.^22^	N/A
DescTools version 0.99.57	Signorell, A.^43^	N/A
lmerTest version 3.1–3	Kuznetsova et al.^44^	N/A
10x Genomics Cell Ranger version 7.0.1	Zheng et al.^45^	https://www.10xgenomics.com/
Clustree version 0.5.1	Zappia and Oshlack^46^	https://github.com/lazappi/clustree
scDblFinder version 1.18.0	Germain et al.^47^	https://github.com/plger/scDblFinder
dittoSeq version 1.16.0	Bunis et al.^48^	https://github.com/dtm2451/dittoSeq

### Experimental model and study participant details

#### Animals

All *in vivo* studies were performed using 8–12 week old female NOD.Cg-Prkdc^SCID^ Il2rg^tm1Wjl^/SzJ (NSG) mice from Charles River. Animals were housed at CRUK Cambridge Institute animal facility, housed in groups of 5 mice per cage, and randomly assigned to experimental groups. All animal work was performed under the Home Office regulatory framework in the UK (project licence number: P1266F82E).

#### Cell lines

HEK293T cells were used for lentiviral packaging. These cells originated from a female fetal kidney, were grown in DMEM media with 2mM L-glutamine and 10% fetal bovine serum (FBS), and incubated at 37°C in a 5% CO_2_ incubator. For cryopreservation, 5% DMSO was added to the full growth media, and cryovials stored in liquid nitrogen vapor phase. To initiate cultures, cryovials were rapidly thawed in a water bath at 37°C, cells washed in phosphate buffered saline (PBS) with 2% FBS, and then resuspended and plated in full growth media. MDA-MB-231 cells were used to generate spike-in barcode controls. These cells originated from a female breast adenocarcinoma, were grown in DMEM media and 10% FBS, and incubated at 37°C in a 5% CO_2_ incubator. All cell lines were routinely tested and confirmed negative for mycoplasma by real-time qPCR. Cell lines were authenticated using short tandem repeat (STR) testing.

#### PDTX models

All PDTX models were established previously (REF) and are of female origin. The site of origin (primary breast adenocarcinoma or metastasis), PAM50 subtype, IntClust subtype, and receptor status are indicated in Table S1. PDTX samples were previously frozen as small fragments in FBS with 10% DMSO. Upon use, they were rapidly thawed in a water bath at 37°C and cells washed in PBS with 2% FBS prior to use. Formalin-fixed paraffin-embedded tissue sections were used for hematoxylin and eosin staining and confirmed to represented breast adenocarcinomas. PDTX models were authenticated using STR testing.

### Method details

#### Barcode library construction and diversity validation

Three barcode libraries (BC1–3, Figure S1A) were constructed by inserting a 27bp semi-random barcode sequence based on a previous design,^49^ along with a 4bp unique library ID sequence, into the 3′ untranslated region of the GFP fluorescence reporter gene of the pLARRY-EGFP vector,^18^^,^^37^ which was a gift from Fernando Camargo (Addgene plasmid # 140025). The plasmid libraries for BC1 and BC2 were sequenced to a depth of 30 and 16 million reads, respectively. To validate the diversity of barcode sequences captured upon transduction of human cells, 10^5^ MDA-MB-231 cells (a human breast cancer cell line), were transduced with the lentiviral libraries BC1 and BC2 each in triplicate. Each replicate was sequenced separately, and the resulting barcode sequences pooled for analysis of barcode diversity. BC1 and BC2 were validated to each contain ∼1 million unique barcode sequences (Table S8), and the pooled distributions of reads corresponding to each unique barcode sequence from sequencing of the plasmid libraries and test transduced cells are shown (Figure S1B). We validated by *in silico* simulation that upon repeated sampling of both barcode libraries, there is a less than 1% chance of any 1,000 randomly selected barcodes having a Hamming distance of 4 or less (Figure S1C), ensuring that the diversity and distribution of barcodes within both libraries were sufficiently diverse for our use. We can therefore be confident that any barcode sequences detected represent unique barcodes, even after grouping reads with a Hamming distance of ≤1 to reduce noise from any potential sequencing errors (Figure S1D). BC1 and BC2 were used for clonal tracking experiments while BC3 was used to generate spike-in controls which served to calibrate read count between samples and to determine clone size in absolute cell number, as previously described^50^ (Figures S1E and S19). Across all multiplexed targeted DNA amplicon sequencing runs for DNA-based barcode analysis, clones were detected with a sensitivity of 95% for clones consisting of 20 cells, and 100% for clones consisting of 50 cells or more after applying thresholds to remove possibly aberrant reads (Figure S1F).

#### Lentiviral packaging and transduction

Lentiviruses were packaged in HEK293T cells in T175cm^2^ flasks using the Lipofectamine 3000 kit according to manufacturer’s protocol (Invitrogen) using psPAX2 and pMD2.G packaging plasmids. psPAX2 and pMD2.G were gifts from Didier Trono (Addgene plasmids # 12260 and 12259). Upon harvesting of the lentiviral particles, the supernatant was concentrated (Takara Bio Lenti-X Concentrator), and flash frozen in single-use aliquots. Titration of lentiviral titer was performed using MDA-MB-231 cells and analyzed by flow cytometry for GFP-positive cells. Each lentiviral transduction was performed in a total volume of 100ul in polystyrene FACS tubes containing a maximum of 10^6^ cells in growth media with 0.8ug/100ul Polybrene and incubated in a CO_2_ incubator at 37°C for 4 h. At the end of this time, the cells were washed 3 times with PBS +5% FBS, and finally resuspended in growth media with 50% matrigel to be immediately implanted into mice. Growth media was RPMI 1640 media with EGF (20 ng/mL), FGF (20 ng/mL), and B-27 supplement (1X concentration). A small aliquot (∼5%) of the transduced cells were plated with growth media into tissue culture plates and allowed to incubate at 37°C for 48–72 h prior to analysis by flow cytometry to determine transduction efficiency based on percentage of DAPI-negative (viable) GFP positive (transduced) cells. Transduction conditions were also optimised to target a transduction efficiency of less than 30% to reduce the likelihood of multiple barcode integrations per cell and transduction efficiency was analyzed by flow cytometry for every xenograft generated. Where different transduction efficiencies were obtained in separate experiments for the same PDTX model, a range is indicated (Table S9). Based on our scRNAseq dataset, an estimate of actual multiple barcode integrations was 2.4%, though this is likely an over-estimate as this value also includes cell doublets from scRNAseq (Table S10).

#### PDTX dissociation and mouse engraftment

PDTX samples were previously frozen as small fragments in FBS+10%DMSO as previously described.^29^ Aliquots of PDTX material were rapidly thawed, washed in cold PBS+2%FBS, and dissociated as per manufacturer’s protocol (Miltenyi Biotec). The single cell suspension was then mouse-cell depleted by magnetic bead separation (Miltenyi Biotec), and viable cells counted prior to lentiviral transduction (as described above). Following lentiviral transduction, cells were resuspended in 100ul of 50% Matrigel and 50% RPMI growth media, and immediately implanted subcutaneously into 8–12-week-old female NSG mice. Mice were then monitored, and tumor growth measured weekly, and tumors harvested prior to reaching the size limit of 1500mm^3^. Upon tumor harvesting, the tumors were diced into small fragments measuring 2–3 mm^3^. These fragments were then mixed and randomly split into 3–5 cryovials and frozen in FBS+10%DMSO to distribute as evenly as possible any clonal heterogeneity from each tumor into separate aliquots. To establish secondary xenografts, frozen vials of tumor fragments were dissociated into a single cell suspension, mouse cell depleted, and then injected into secondary mice. All 4 PDTX models were passaged into secondary mice at the same time. Time to tumor harvest ranged from 38 to 202 days and 84–180 days for primary and secondary xenografts, respectively. While every effort was made to allow tumors to reach the size endpoint, issues such as skin ulceration required earlier endpoints. The time to tumor harvest related to cell dose implanted is represented in Figure S3 and tumor size at time of harvest is reported in Table S1. For subsequent DNA sequencing analysis, 1–2 of these vials containing multiple small fragments of viably frozen PDTX material was dissociated into a single cell suspension, mouse cell depleted (as described above), the number of viable cells counted, and a maximum of 2x10^5^ cells per tumor taken for gDNA extraction and subsequent PCR amplification for barcode DNA amplicon sequencing. The proportion of the entire tumor sampled for PCR amplification, and corresponding limit of detection for individual clones for each xenograft model is presented in Table S11. Limit of detection = 20 cells (corresponding to the sensitivity for clone detection of 95% for clones containing 20 cells or more, Figure S1) x (100/percent of tumor sampled). For scRNAseq, the cells were additionally sorted on the flow cytometer by forward and side scatter to exclude debris, and further selection for DAPI-negative viable cells. All xenograft models used in this study were established and previously published elsewhere.^29^^,^^51^ All animal work was performed under the Home Office regulatory framework in the UK (project licence number: P1266F82E).

#### Barcode DNA amplicon sequencing

Multiplexed amplicon sequencing was achieved by amplifying purified gDNA from cells (maximum of 2x10^5^ cells per sample using the prepGEM Universal DNA extraction kit, ForenteQ Limited) or purified lentiviral plasmid DNA (50ng per sample) with one of three pairs of staggered primers containing an i7 and i5 linker sequence combined with a target-specific sequence (underlined below) known to flank the barcode sequence. The target-specific sequence was staggered around the barcode sequence to increase library complexity and improve cluster recognition on the Illumina MiSeq.

Pair 1 Forward primer: TCGTCGGCAGCGTCAGATGTGTATAAGAGACAG**TAGAAGGCACAGGTCGACAG**.

Pair 1 Reverse primer: GTCTCGTGGGCTCGGAGATGTGTATAAGAGACAG**GTCTAGACTCACTGGCCGTC**.

Pair 2 Forward primer: TCGTCGGCAGCGTCAGATGTGTATAAGAGACAG**GCAACTAGAAGGCACAGGTC**.

Pair 2 Reverse primer: GTCTCGTGGGCTCGGAGATGTGTATAAGAGACAG**GACTCACTGGCCGTCGTTTT**.

Pair 3 Forward primer: TCGTCGGCAGCGTCAGATGTGTATAAGAGACAG**CAACTAGAAGGCACAGGTCG**.

Pair 3 Reverse primer: GTCTCGTGGGCTCGGAGATGTGTATAAGAGACAG**AGACTCACTGGCCGTCGTTT**.

Following a 1^st^ PCR amplification using the above primers and Q5 high-fidelity DNA polymerase (New England Biolabs) at an annealing temperature of 67°C for 25 cycles, the product was ethanol precipitated, and amplified in a 2^nd^ PCR reaction containing the IDT for Illumina UD Indexes (Illumina) for 5 cycles at an annealing temperature of 62°C. The final product was size-selected, ethanol precipitated and quantified by qPCR using the KAPA Library Quantification kit according to manufacturer’s protocol (Roche). Each multiplexed library was pooled in equimolar ratio and sequenced on the Illumina MiSeq for 75bp paired-end sequencing. On average each sequencing run yielded 2 x 10^7^ paired-end reads, resulting in between 5 x 10^5^ to 1 x 10^6^ paired-end reads per sample analyzed.

#### Barcode sequence data processing

Multiplexed DNA amplicon sequencing was performed on the Illumina MiSeq and demultiplexed into individual FASTQ files. These files were run through FastQC (version 0.11.9)^38^ as an initial quality control step. Barcode reads were retrieved using cutadapt (version 1.10)^39^ to trim flanking constant sequences, match the exact required length of 31bp, and filter reads with a Phred quality score of <30. Barcode reads not matching the exact expected pattern and sequence of constant and random nucleotide sequences were removed. The remaining reads were then grouped if they had an edit distance of 1 or less, and counted to provide the number of reads for each unique barcode sequence detected, and separated based on whether the reads were from BC1, BC2, or BC3 (Figure S1D). Reads for BC3 corresponded to the spike-in cell controls and were thus used to calculate the fractional read value (FRV). FRV = [reads for clone of interest]/[sum of reads from all spike-in controls]. These spike-in controls served as technical replicates ranging between 10 cells to 12,500 cells with between 24 and 308 technical replicates each, depending on the cell dose (Figure S1F). Using the FRV for all the spike-in controls, a correlation was derived between log10(FRV) and log10(cell number)(Figures S1E and S19). Using this correlation, the calculated FRV for experimentally detected barcode clones from BC1 or BC2 was used to calculate the clone size in absolute cell number allowing us to correct for any amplification bias that may be introduced during library preparation for sequencing, as previously published.^50^

#### Bulk RNA sequencing and data analysis

RNA was extracted from flash frozen PDTX material using TRIzol (Invitrogen) according to manufacturer’s protocol. RNA concentration was measured on the Qubit 4 (ThermoFisher Scientific), and RNA quality was assessed on the Agilent 4200 TapeStation. RNA sequencing libraries were prepared by the CRUK Genomics Core Facility using the TruSeq Stranded mRNA library preparation kit (Illumina), according to manufacturer’s protocol, and 100 bp paired end sequencing performed on the Illumina NovaSeq 6000 S1 flowcell. Raw sequence reads were subjected to quality control, and Trimmed Mean of M-values normalization. As expected, principal component analysis revealed samples belonging to the same PDTX model clustered together (Figure S4). Differential gene expression analysis was performed using the glmQLFit function from edgeR (version 3.32.1),^40^ with a quasi-likelihood negative binomial generalized log-linear model. Adjusted *p*p-values were calculated using the Benjamini-Hochberg method to control for false discovery rate.^52^

#### scRNAseq and data processing

To prepare samples for scRNAseq, viably frozen tumor fragments were dissociated into a single cell suspension as described above, including mouse cell depletion. Viable cells were then sorted by FACS based on exclusion of DAPI, and a subset of samples were enriched for GFP positive cells to increase the yield of barcode clones detected by scRNAseq (Table S12). 10X Chromium scRNAseq was performed using standard 3′ v3.1 chemistry or 3′ v3.1 HT chemistry to target recovery of 1x10^4^ and 2x10^4^ cells, respectively, prior to sequencing on the Illumina NovaSeq S4 flowcell targeting 2x10^4^ reads per cell. For data processing, the demultiplexed FASTQ files were processed by *cellranger* (v7.0.1)^45^ count command, and a QC threshold applied to cells based on the number of UMIs and percentage mitochondrial UMIs (Table S2). A count matrix was then created from the remaining cells, and the matrices from all samples were combined to a single count matrix. The *metacell* R package (version 0.3.7)^23^ was used to partition cells into small groups termed metacells that represent unique transcriptional cell states. The feature genes used to generate the metacells were first selected by their strong expression and high variability and we then removed blacklisted genes (mitochondrial and a few strong non-coding genes, and gene modules correlated with cell cycle, hypoxia, interferon and stress responses, see Table S13). This resulted in a set of 4,269 feature genes that were used in the creation of metacells (Table S14). Metacells were derived as previously described^23^, using K = 250 and standard bootstrapping. The derived final model included 167,375 cells partitioned into 1,107 metacells. The mean 151 cells per metacell in our dataset is expected and consistent with prior studies.^23^^,^^53^ Notably, barcode clone identity was not a factor used in the partitioning of metacells. Barcode reads were then extracted from the same demultiplexed FASTQ files similar to what was done for multiplexed DNA amplicon sequencing described above. When ≥2 lentiviral barcodes were associated with a single cell and this pattern was not repeated in multiple cells, these were identified as likely cell doublets from the scRNAseq workflow, and those barcodes and gene expression profiles were blacklisted and excluded from downstream analysis. However, when the same pattern of ≥2 lentiviral barcodes were detected in multiple single cells, this was identified as likely multiple integrations per cell of origin, and all but one of these barcodes were blacklisted so as not to count the same clone more than once. We performed scRNAseq in 3 main batches. The partitioning of cells to metacells use feature genes to measure cell-cell similarity, and removes genes that are known to be affected by technical effects from the feature genes. The genes removed are specified in Table S13. As indicated in Table S2, for STG139, P1, S1 and S2 xenografts were part of the same sequencing batch. For STG201, IC07 and AB040, P1 was in a separate sequencing batch from S1-S3 xenografts. As shown in Figure S8 where the dominant propagating clones are compared across xenografts, cells were found to be partitioned into metacells by model and not by batch. As such, we did not need to perform any batch correction in our analysis. *Seurat* (v5.1.0)^27^ was also used to cluster and visualize the generated count matrix by transcriptional state (see Table S15 for clustering parameters). Cluster stability was optimized using the R package *Clustree* (v0.5.1),^46^ and cluster composition was quality checked using the R package *scDblFinder* (v1.18.0)^47^ to identify doublet clusters and visualized using the R package *dittoSeq* (v1.16.0).^48^

#### scRNAseq differential gene expression analysis

The cell states identified from *metacell* R package (version 0.3.7)^23^ were used for further downstream analysis. This included pseudobulk differential gene expression analysis by cell level UMI downsampling, pooling cells in each compared group to a single pseudobulk profile and then normalising the total number of UMIs in both profiles. Genes expressed in at least 50% of cells in the enriched group and at least 0.1 mean UMI per cell were considered. P-values per gene enrichment were calculated by Mann-Whitney test and corrected by the FDR method. We performed gene set enrichment analysis with *pathfinder* (version 2.3.0.9000)^42^ using the Hallmark gene set (downloaded from the Human Molecular Signatures Database, MSigDB). Calculation of Hallmark gene signature scores for every single cell in the scRNAseq dataset was performed using *UCell* R package (v2.2).^41^ All Hallmark gene sets were obtained from MSigDB. The mesenchymal signature was taken from the epithelial to mesenchymal transition Hallmark gene set, and the epithelial signature was obtained from a previously published epithelial gene set associated with an epithelial state in HMLER transformed human mammary epithelial cells.^54^

#### Derivation of gene signatures for epithelial cell phenotypes

Epithelial cell signature genes (Table S16) were derived from a scRNAseq dataset that profiled healthy breast tissues.^26^ We re-analysed this dataset with the *metacell* package and identified the 3 main normal epithelial cell types the authors reported – Basal, LP and ML. We then extracted differentially expressed genes by comparing the cells from each cell type against cells from the remaining two cell types, and removed genes that were enriched in more than a single cell type.

#### Transcriptional similarity analysis from scRNAseq dataset of single cell-derived clones

To analyze the dynamics of transcriptional processes within clones as they evolved in the absence of subclonal lineage recording data, we used transcriptional similarity to order metacells comprising each dominant propagating clone. In this way, we assume that phenotypic changes are gradual and a cell will be transcriptionally similar to its parent. We note that abrupt transcriptional changes might occur and that cells can switch back and forth between transcriptional states, so our ordering of metacells is putative. Using this approach, we used the metacell similarity graph (Figures 6A and S17A), in which the 3 most transcriptionally similar metacells are connected. The root cell states (marked as distance 0) were defined as the metacells containing at least 3 cells from the primary xenograft, suggesting these cell states represent the early transcriptional profile of the clone. We traversed the metacell similarity graph to mark each metacell by its transcriptional distance on the graph from the nearest root. Ultimately, 8 root metacells were identified for STG139 and 3 for STG201. We then used the ordered groups of metacells to explore transcriptional dynamics within the dominant propagating clones. To follow these transcriptional changes, we clustered strong and highly variable genes per model, and defined 18 clusters for STG139, and 11 for STG201 that we termed gene modules (GMs, Tables S17 and S18). We then examined the change in expression of these GMs across the transcriptional distances. The GMs we highlighted in Figures 6 and S15–S18 were selected based on containing genes related to epithelial and mesenchymal transition. The other GMs identified are reported in Tables S17 and S18, but data not shown. While grouping of metacells by order cannot be perfect, nor validated without direct subclonal lineage tracking, this analysis highlights the relative changes between the GMs within a dominant propagating clone.

### Quantification and statistical analysis

#### *In silico* simulation of clone detection

To consider whether the negative correlation between CIC frequency and the number of cells implanted was due to a technical artifact resulting from sampling proportionately fewer barcodes in xenografts established from more barcoded single cells (and thus represent a more diverse barcode pool), we simulated the expected number of detected clones per sample. In each round of the simulation, we started with the actual number of cells implanted that had detectable barcodes (based on the percentage of GFP-positive cells in the sample). We then allowed all cells to double until reaching the target number of cells (mean number of estimated cells by tumor size). Barcodes were then sampled based on the number of reads sequenced for the sample and then the number of unique barcode clones detected were counted. Sampling was done with repetitions to represent PCR amplification. 50 repetitions were performed for each sample. The number of unique barcode clones from this random sampling was used to calculate CIC frequency and plotted against the number of cells implanted in each experiment (Figure S20). This negative correlation suggested that there is a sampling effect related to cell dose (and thus diversity of the barcode pool). This was used to correct the experimentally observed correlation by subtracting the slope of the negative correlation from simulated data from the slope of the negative correlation from experimental data. A significant negative correlation remained, suggesting there is a co-existing true biological effect pertaining to the suppression of clonogenic activity in the presence of more starting cells.

#### *In vivo* clone doubling times

*In vivo* clone doubling times for experimentally detected clones were calculated as time *in vivo* (in days) divided by log_2_(absolute clone size). We analyzed the density distribution of *in vivo* doubling times by merging the data from all clones across all PDTX models and then fitted a mixture Gaussian model to all 19,303 primary clones using the *mclust 5* R package.^22^ To determine whether the distinct peaks observed in the density distribution of *in vivo* doubling times were consistent with the hypothesis of groups of clones with different growth rates or with common stochastic variability, we performed an *in silico* simulation of clone doubling times. For each PDTX model, we analyzed the density distribution of *in vivo* doubling times and fitted a mixture Gaussian model using the *mclust 5* R package.^22^ The number of components was determined by utilising the Akaike Information Criterion. Each component of the mixture model represents a subpopulation of clones with a given mean doubling time. We used the estimated mean doubling times (μ) from each component of the mixture model as the basis for our simulation. The proportion of clones belonging to each component (p) in the experimental data was used to determine the initial size of each simulated subpopulation. In the simulation, we started with a population of cells comprising each subpopulation identified by the mixture model. For each clone, we simulated its growth over time using an exponential distribution with rate parameter $=\frac{1}{\mu }$ , where μ is the mean doubling time for that subpopulation. This approach allows for stochastic variation in doubling times around the mean for each subpopulation. These cells were allowed to divide over time at varying doubling times until reaching 10^7^ total cells, simulating the maximum allowable tumor size in mice. The resulting distribution of doubling times from this simulated population was then compared to the experimental data. The sum of cells obtained for each clone follows a gamma distribution, which, when the number of cells is large, asymptotically approaches a normal distribution. This mimics the mixture model observed in the clone doubling times. By comparing the simulated distribution to the experimental data, we can confirm whether the distinct peaks observed in the experimental data are indeed due to biologically distinct subpopulations with different growth rates, rather than individual stochastic variability alone.

Model: for each subpopulation

(1) The model begins with an initial cell size for each clone 1$N_{0}=$.
(2) The subpopulation at each time step t is given by $N_{t}=2^{r_{1}+r_{2}+\cdots +r_{t}}$, where $r_{i}\;∼\;\exp\left(\lambda \right)$ is an exponential random variable with rate parameter λ.

Taking the logarithmic transformation returns: $\log_{2}\left(N_{t}\right)=r_{1}+r_{2}+\cdots +r_{t}$

With distribution: $\log_{2}\left(N_{t}\right)\;∼\;Gamma\left(t,1/\lambda \right)\text{.}$

Limit distribution under central limit theorem.

(1) As t→∞, $\frac{\log_{2}\left(N_{t}\right)}{t}$ converges to a normal distribution.
(2) The resulting distribution is $\frac{\log_{2}\left(N_{t}\right)}{t}\;∼\;N\left(\frac{1}{\lambda },\frac{1}{t\lambda^{2}}\right)$.

This process was conducted separately for each of the 26 PDTX models. At the end of the simulation, the resultant cell population was used to calculate the *in vivo* doubling time of the various cell populations (time stop set as 10^3^ days, cell max capacity of 10^7^ cells), and the density plot of this population distribution was compared against our experimental data for each of the 26 PDTX models.

#### Tumor volume measurements

To assess the size of the tumors in mice, calipers were employed on a weekly basis to record the dimensions, specifically the height (h), and width (w). The tumor volume (mm^3^) was determined by using the equation:

$$Tumour\;volume=\frac{w^{2}h}{2}$$

#### Linear mixed effect model analysis

Model random slope and intercept:

$$\log_{10}\left({CIC}_{frequencyij}\right)=\beta_{0}+\beta_{1}*\log_{10}\left({Cell}_{simplantedij}\right)+\left(u_{0j}+u_{1j}*\log_{10}\left({Cell}_{simplantedij}\right)\right)+\epsilon_{ij}$$

Here, $\beta_{0}$ is the global intercept (fixed effect), $\beta_{1}$ is the global coefficient (fixed effect) for the log-transformed number of cells implanted. While, $u_{0j}$ is the random intercept for the j-th model (PDTX) and $u_{1j}$ is the random slope for the j-th level model (PDTX), representing how the effect of “$\log_{10}\left({Cells}_{implanted}\right)$” varies across different models (PDTXs), and ϵ is the residual error for the i-th observation in the j-th model (PDTX).

Likelihood ratio test:

To assess whether the random slopes in the mixed effects model are significant, we performed a likelihood ratio test comparing the model random slope and intercept and a model without random slopes using *lmerTest* R package version 3.1–3.^44^

Model with only random intercepts:

$$\log_{10}\left({CIC}_{frequencyij}\right)=\beta_{0}+\beta_{1}*\log_{10}\left({Cells}_{implantedij}\right)+u_{0j}+\epsilon_{ij}$$

Then comparing the previous two models using an ANOVA test, we get a *p*p-value of 0.008065.

#### Statistics

We employed a chi-squared test of independence to assess the relationship between clone doubling time classification (Fast, Medium, Slow) and biological categories such as PAM50 subtype (Figure 3C left, *p*p-value <2.2 x 10^−16^), IntClust subtype (Figure 3C middle, *p*p-value <2.2 x 10^−16^), and comparison of propagating versus transient clones (Figure 3C right, *p*p-value <2.2 x 10^−16^). We performed an unpaired student’s t-test to assess the statistical significance of the difference in Hallmark gene signature expression from scRNAseq data (Figures 5C and 5D).

#### Entropy

We calculated entropy using the Shannon index with R package *DescTools* version 0.99.57.^43^ Entropy was calculated per clone, based on the fraction of cells the clone contributed to each metacell. Notably, it was not possible to calculate entropy for models STG139 and IC07 since each of these models only have one propagating clone with minimal contribution from transient clones to the various metacells within the PDTX model. Entropy was also calculated per metacell, based on the fraction of cells contributed to each metacell by different clones.

## Supplemental information

Document S1. Figures S1–S20 and Tables S2, S8–S12, and S15

Table S1. Summary of CIC frequency in 26 PDTX models assayed Shown is the information for each PDTX model assayed, including PAM50 subtype, hormone receptor status, and integrated cluster (IntClust) subtype. The CIC frequency was calculated at each cell dose tested per model, and the 95% confidence interval of CIC frequency indicated. Source indicates whether the PDTX model was initially established from patient material that came from the primary tumor (P) or site of metastasis (M). UND, undefined; NR, not recorded; NA, not applicable

Table S3. Differentially expressed genes comparing cells from fractions 1 and 2 in basal model STG139

Table S4. Differentially expressed genes comparing cells from fractions 1 and 2 in basal model STG201

Table S5. Differentially expressed genes comparing cells from propagating and transient clones in basal model STG139

Table S6. Differentially expressed genes comparing cells from propagating and transient clones in basal model STG201

Table S7. Differentially expressed genes comparing cells from propagating and transient clones in luminal B model AB040

Table S13. List of genes blacklisted from creation of metacells

Table S14. List of feature genes used in the creation of metacells

Table S16. List of genes corresponding to the basal, LP, and ML gene signatures derived from normal human mammary epithelial cell types

Table S17. Genes and transcription factors corresponding to the defined GMs for STG139

Table S18. Genes and transcription factors corresponding to the defined GMs for STG201

Document S2. Article plus supplemental information
