# Supplementary material for: Fitness and transcriptional plasticity of human breast cancer single-cell-derived clones
Source: Cell Rep. 2025 May 12;44(5):115699. doi: 10.1016/j.celrep.2025.115699 (PMC12117018; doi:10.1016/j.celrep.2025.115699)
Supplement: Document S1. Figures S1–S20 and Tables S2, S8–S12, and S15 [file mmc1.pdf]

Cell Reports, Volume *44*

Supplemental information

Fitness and transcriptional plasticity of human breast cancer single-cell-derived clones

Long V. Nguyen, Yaniv Eyal-Lubling, Daniel Guerrero-Romero, Sarah Kronheim, Suet-Feung Chin, Raquel Manzano Garcia, Stephen-John Sammut, Giulia Lerda, Allan J.W. Lui, Helen A. Bardwell, Wendy Greenwood, Hee Jin Shin, Riccardo Masina, Katarzyna Kania, Alejandra Bruna, Elham Esmaeilishirazifard, Emily A. Kolyvas, Samuel Aparicio, Oscar M. Rueda, and Carlos Caldas

DOCUMENT S1

SUPPLEMENTARY FIGURES

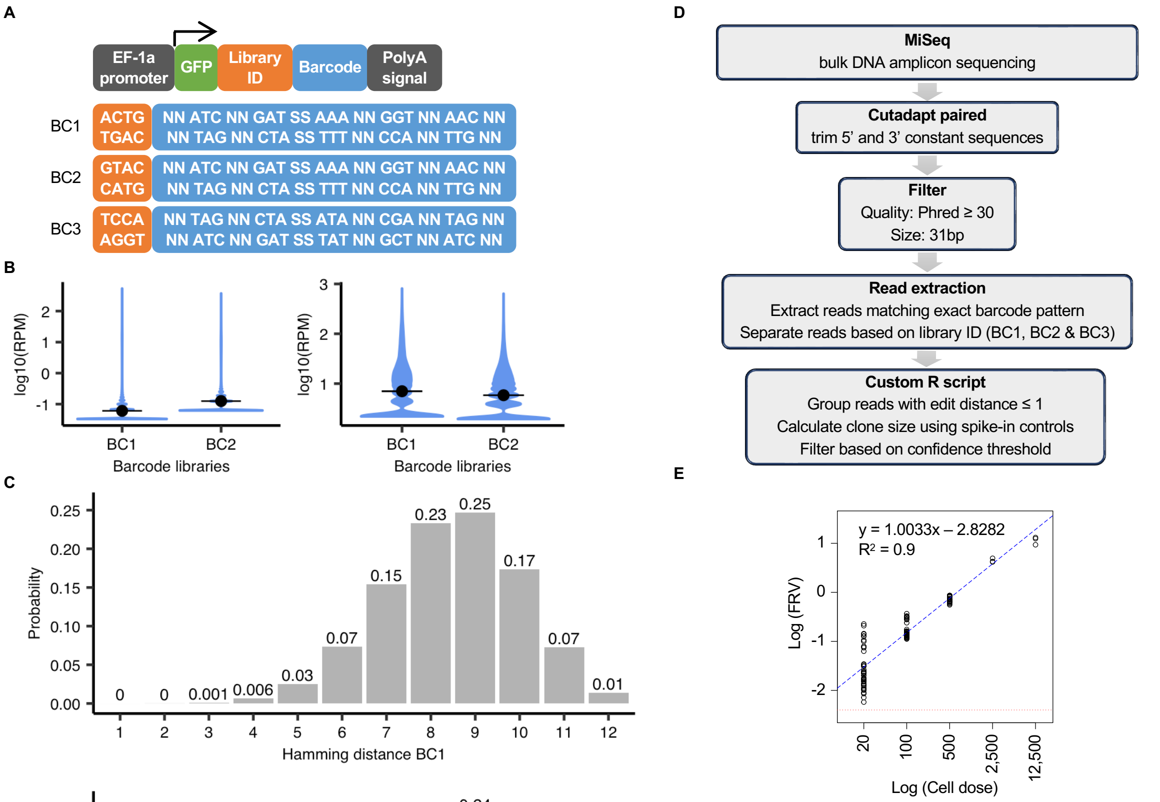

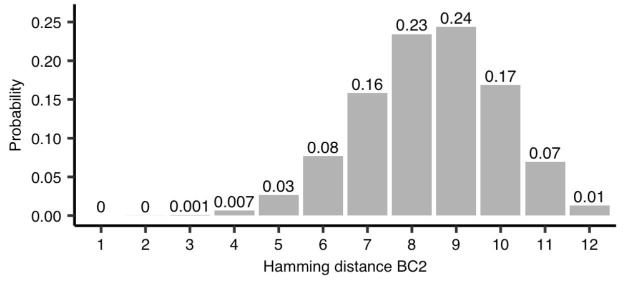

F

Spike-in control (cells)	Detected by sequencing	Detected above threshold
12,500	24 / 24 (100%)	24 / 24 (100%)
10,000	30 / 30 (100%)	30 / 30 (100%)
2,500	24 / 24 (100%)	24 / 24 (100%)
1,000	30 / 30 (100%)	30 / 30 (100%)
500	308 / 308 (100%)	308 / 308 (100%)
100	308 / 308 (100%)	308 / 308 (100%)
50	30 / 30 (100%)	30 / 30 (100%)
20	275 / 278 (99%)	263 / 278 (95%)
10	26 / 30 (87%)	0 / 30 (0%)

Figure S1. Lentiviral barcode library validation and quantitative clonal analysis approach
(A) Schematic of the three lentiviral vectors from which the barcode libraries were produced: BC1, BC2 and BC3.
(B) The plasmid libraries for BC1 and BC2 were sequenced to a depth of 30 and 16 million reads, respectively, and the distribution of reads corresponding to each unique barcode sequence is shown (left). A human breast cancer cell line MDA-MB-231 was transduced with barcode libraries BC1 and BC2 at 10^5 cells each in triplicate. The pooled distribution of reads corresponding to each unique barcode sequence from the transduced cells is shown (right).
(C) An *in silico* simulation showing the density distribution of calculated Hamming distance between 10^3 randomly selected barcode sequences from BC1 (top) and BC2 (bottom) sampled $5x10^4$ times. This shows a <1% chance of any two barcodes randomly selected having a Hamming distance of 4 or less.
(D) Overall workflow for computational processing of barcode data from amplicon sequencing.
(E) Example log-log relationship between input cell dose per clone and fractional read value (FRV, i.e., read count normalization between multiplexed samples) allowing for experimental clone size to be calculated based on normalized read count.
(F) Sensitivity of clone detection from known spike-in controls with and without the signal-to-noise threshold applied, below which there can be false positive clone detection.

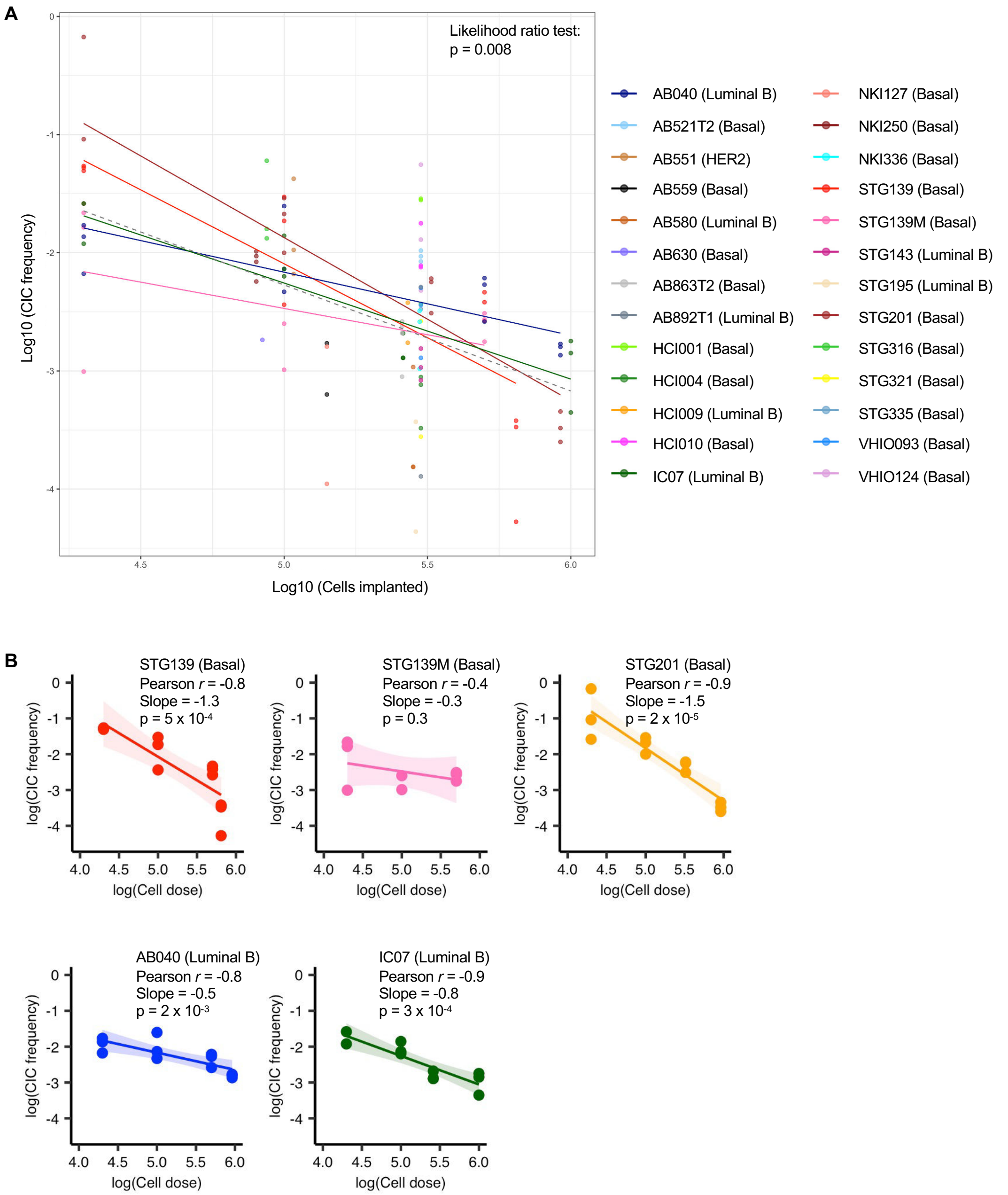

Figure S2. Negative association between number of cells implanted and CIC frequency

(A) For all 110 barcoded tumours, the CIC frequency (y-axis) is plotted against the number of input cells (x-axis) on a log-log plot. Model-specific trendlines are shown in solid colours, for the 5 models with multiple input cell doses (STG139, STG139M, STG201, AB040, and IC07). A linear mixed-effect model (dashed line) shows the overall slope incorporating all 110 barcoded tumours from 26 different PDTX models.

(B) A negative log-log association between cell dose and CIC frequency is shown across 5 PDTX models for which multiple cell doses were tested. Linear models for each model were fitted, and p-values for slope and Pearson correlation are reported.

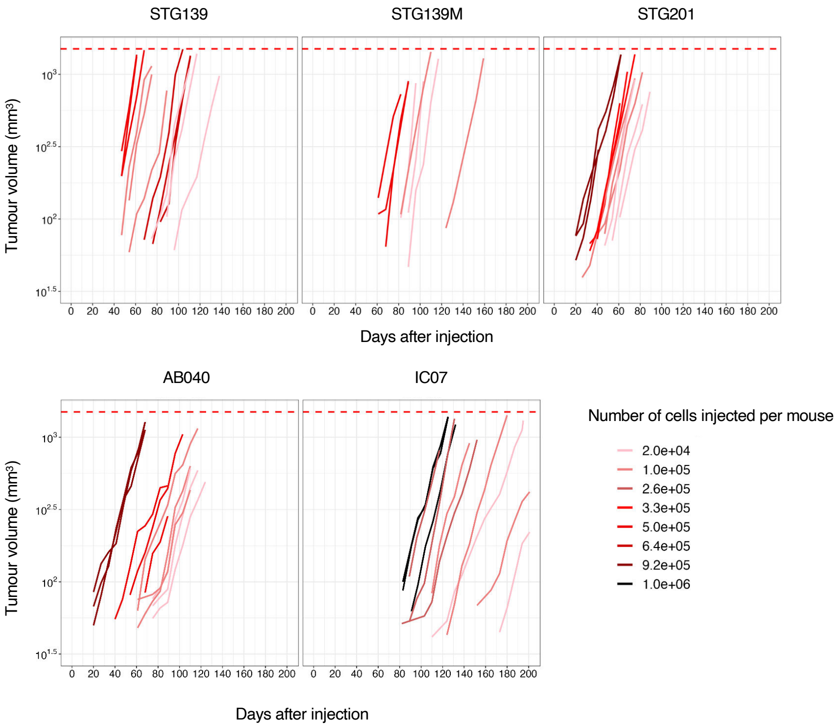

Figure S3. Tumour growth curves for 5 PDTX models for which multiple cell doses were implanted
Tumour growth curves are shown on a log10 scale (y-axis). The growth curves are coloured by input cell dose as indicated in the legend on a scale of dark (highest cell doses) to light (lowest cell doses) red. Tumours are generally harvested before the humane endpoint of 1500 mm^3.

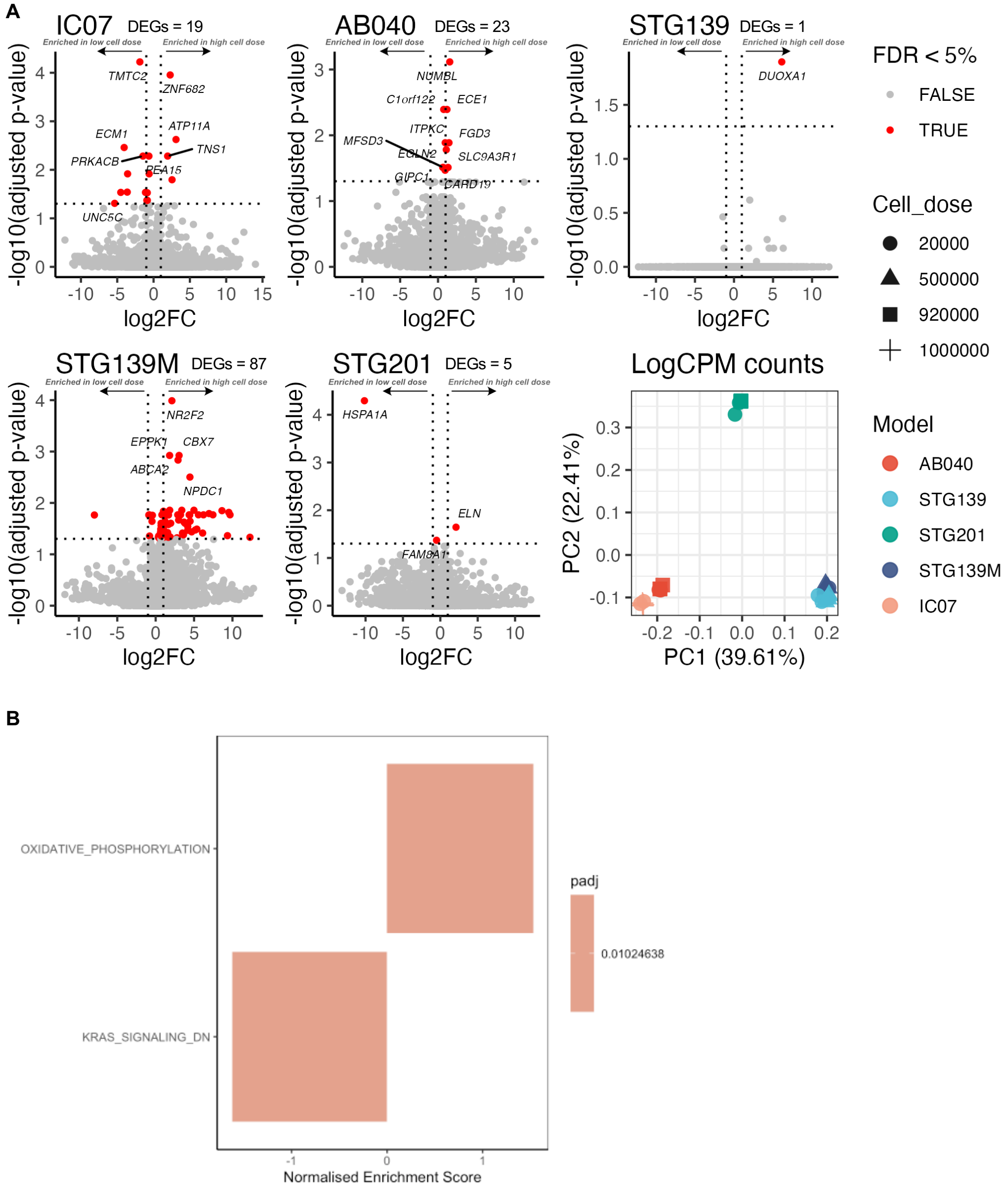

Figure S4. Bulk RNA sequencing of xenografts established from different cell doses

(A) Bulk RNA sequencing was performed on two luminal B barcoded PDTX models (AB040 and IC07) in triplicate at a high cell dose (9.2×10^5 and 1×10^6 cells, respectively) and a low cell dose (2×10^4 cells for both models). Bulk RNA sequencing was also performed on basal barcoded PDTX models (STG139, STG139M and STG201) in triplicate at a high cell dose (5×10^5, 5×10^5, and 9.2×10^5 cells, respectively) and a low cell dose (2×10^4 cells for all three models). Volcano plots from the bulk sequencing differential expression analysis are shown comparing the high cell dose versus the low cell dose for each PDTX model. Differentially expressed genes (DEGs) are indicated in red. Where few DEGs were found, this indicates that the bulk gene expression profiles were similar in tumours established with variable cell doses. This is further substantiated from principal component analysis for these bulk RNA sequencing datasets showing that the samples cluster by model with minimal differences observed by cell dose.

(B) Pathway enrichment analysis for DEGs in basal model STG139M.

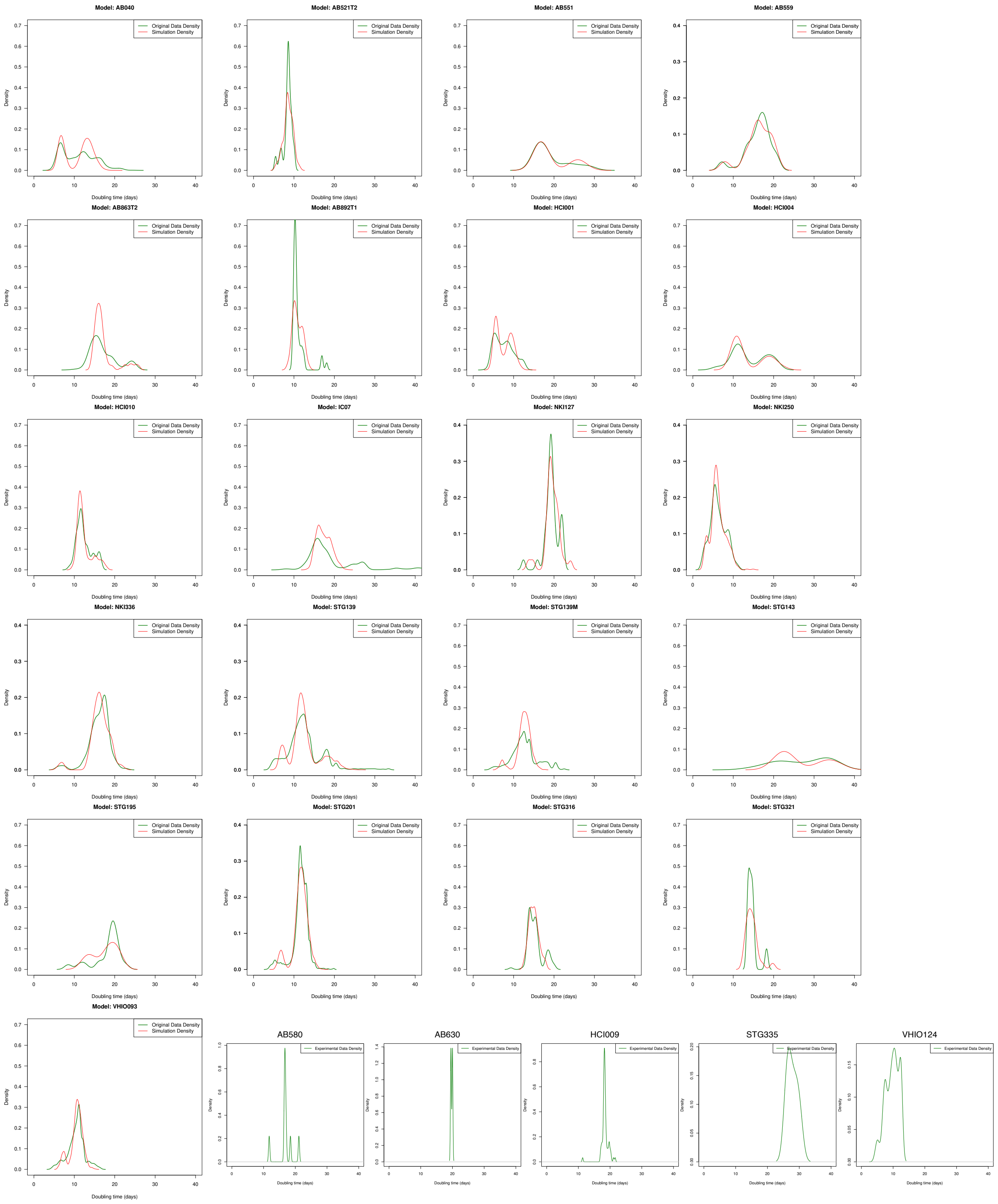

Figure S5. ***In silico*** **simulation of** ***in vivo*** **clone doubling time for all 26 PDTX models.** Shown are density distribution plots of in vivo doubling time for experimentally acquired data (in green), and *in silico* simulated data (in red). For 5 PDTX models (bottom) where the number of clones detected were too few or variable to be modelled with our *in silico* simulation, only the experimental data is shown.

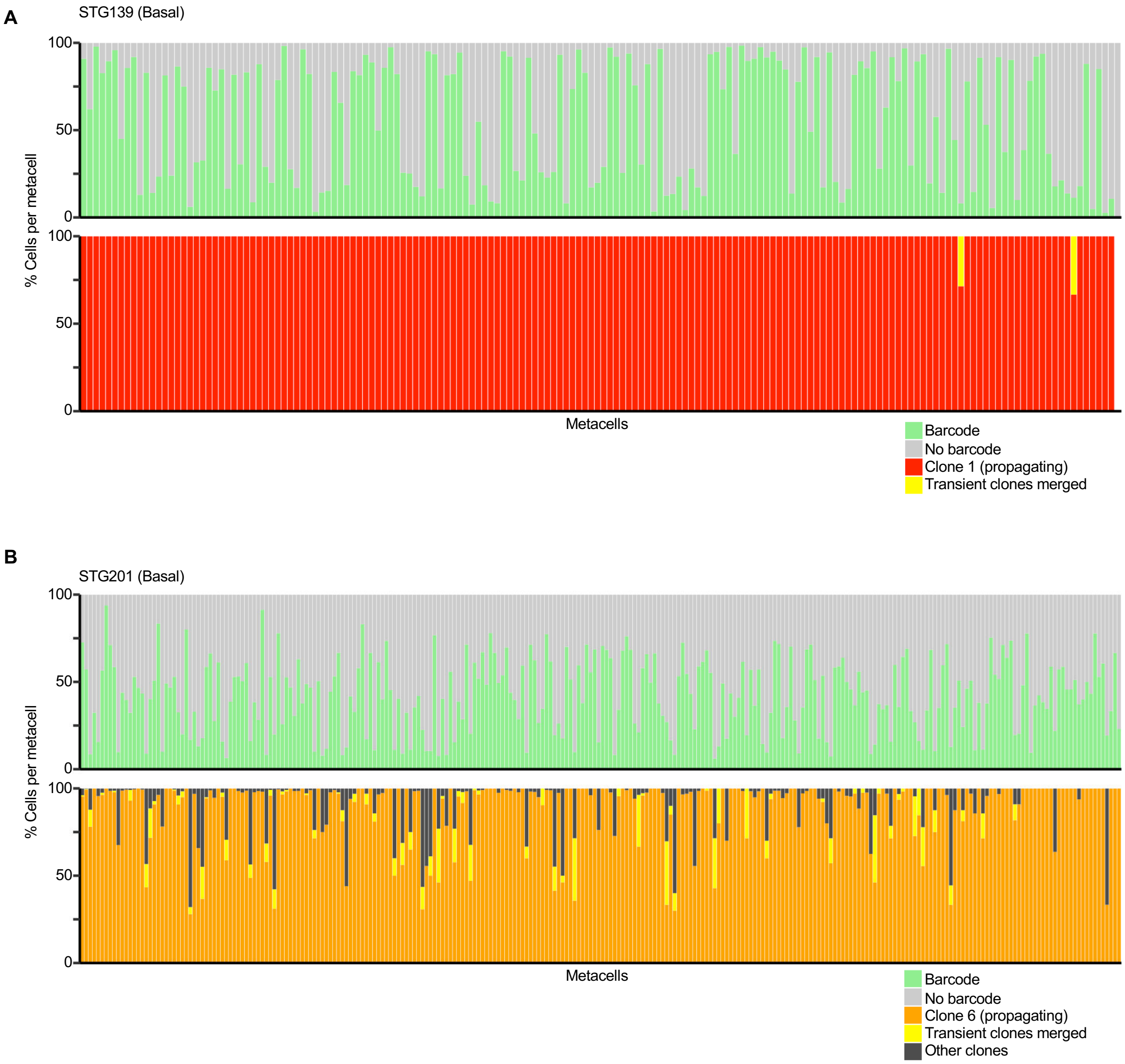

Figure S6. Representation of cells and clones across metacells for basal PDTX models.
(A) Top: stacked barplot showing the percentage composition per metacell of barcoded cells (green) and non-barcoded cells (gray) in basal PDTX model STG139. Bottom: stacked barplot showing the percentage composition per metacell of total barcoded cells from dominant propagating Clone 1 (red), and cells aggregated from all transient clones (yellow). Metacells from left to right in decreasing order of size (i.e., metacells composed of the most to least number of cells), and the ordering is consistent between the top and bottom barplots.
(B) Same as (A) except for basal PDTX model STG201 where the dominant propagating clone is Clone 6 (orange). Non-dominant propagating clones are represented in dark gray.

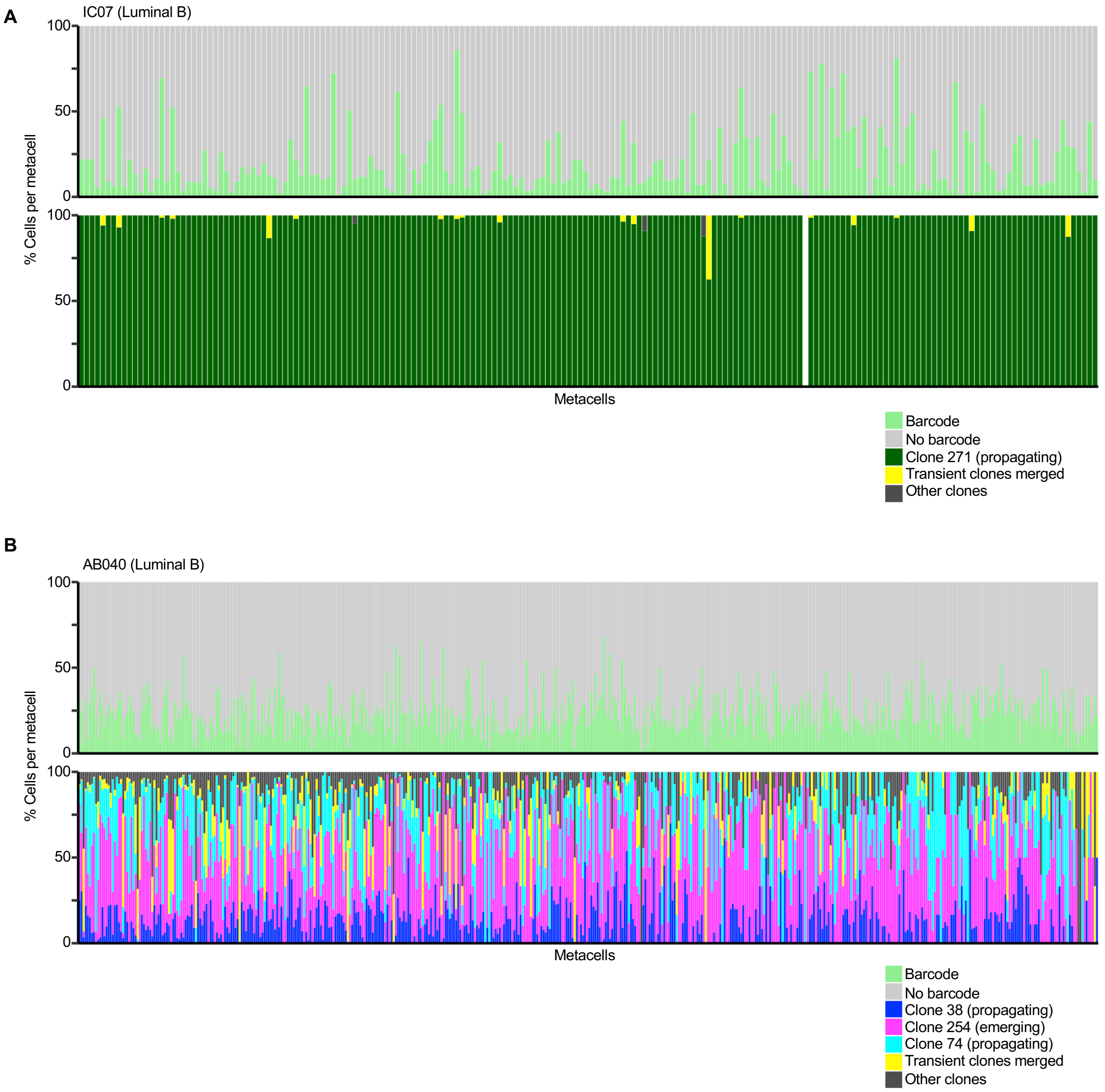

Figure S7. Representation of cells and clones across metacells for luminal B PDTX models.

(A) Top: stacked barplot showing the percentage composition per metacell of barcoded cells (green) and non-barcoded cells (gray) in luminal B PDTX model IC07. Bottom: stacked barplot showing the percentage composition per metacell of total barcoded cells from dominant propagating Clone 271 (dark green), and cells aggregated from all transient clones (yellow). Non-dominant propagating clones are represented in dark gray. Metacells from left to right in decreasing order of size (i.e., metacells composed of the most to least number of cells), and the ordering is consistent between the top and bottom barplots.

(B) Same as (A) except for luminal B PDTX model AB040 where the dominant clones are Clone 38 (blue), Clone 254 (magenta) and Clone 74 (cyan) in secondary replicate xenografts S1, S2 and S3, respectively.

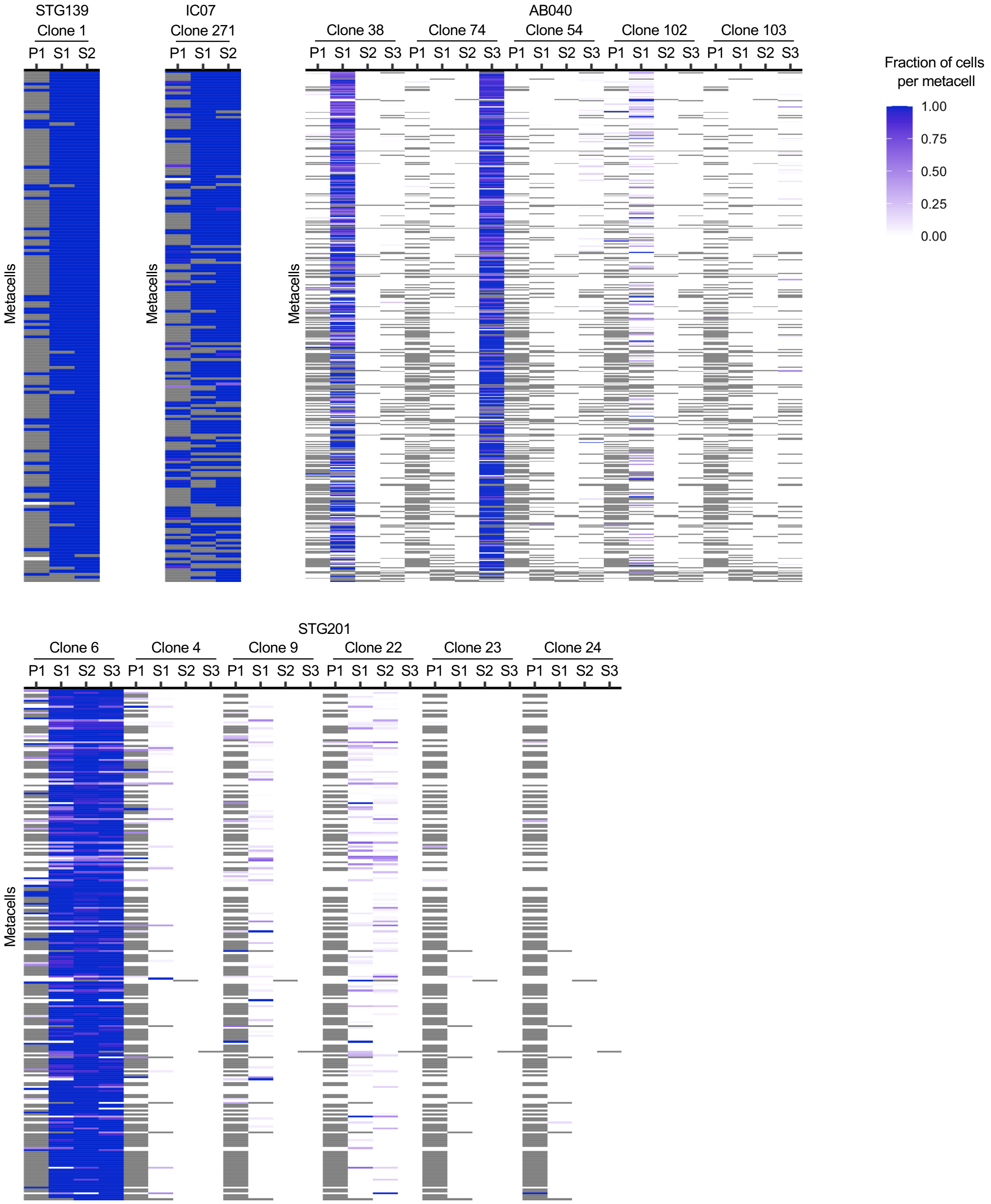

Figure S8. Heatmaps of propagating clone contribution across metacells.
Metacells are ordered from top to bottom in decreasing order of size (i.e., metacells composed of the most to least number of cells). A gray bar indicates when a xenograft did not have any barcoded cells in a metacell. A gradient of white to blue indicates that there are barcoded cells contributing to a metacell on a scale of no cells (white) or all cells (blue) being from that particular propagating clone.

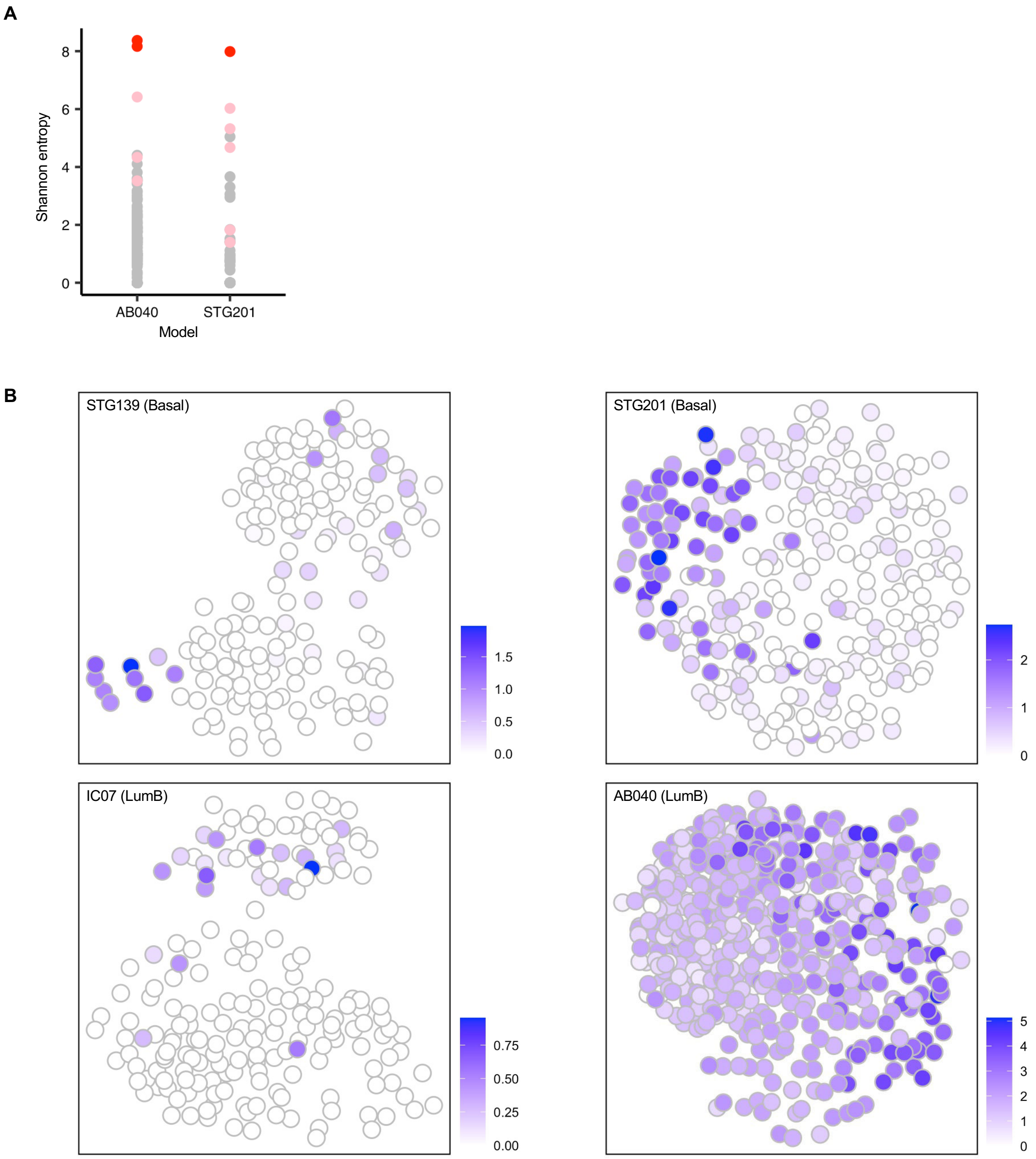

Figure S9. Entropy calculation across clones and metacells.

(A) Entropy calculation using the Shannon index for all clones in luminal B model AB040 and basal model STG201, coloured by type of clone. Notably, entropy could not be calculated for clones in luminal B model IC07 and basal model STG139 because there was only one dominant propagating clone with too little diversity of clonal contribution from transient clones across metacells.

(B) Two-dimensional representation of each metacell-defined cell state for each of the four PDTX models. The metacells are coloured by entropy, where a higher value indicates more diversity of clone representation in a metacell.

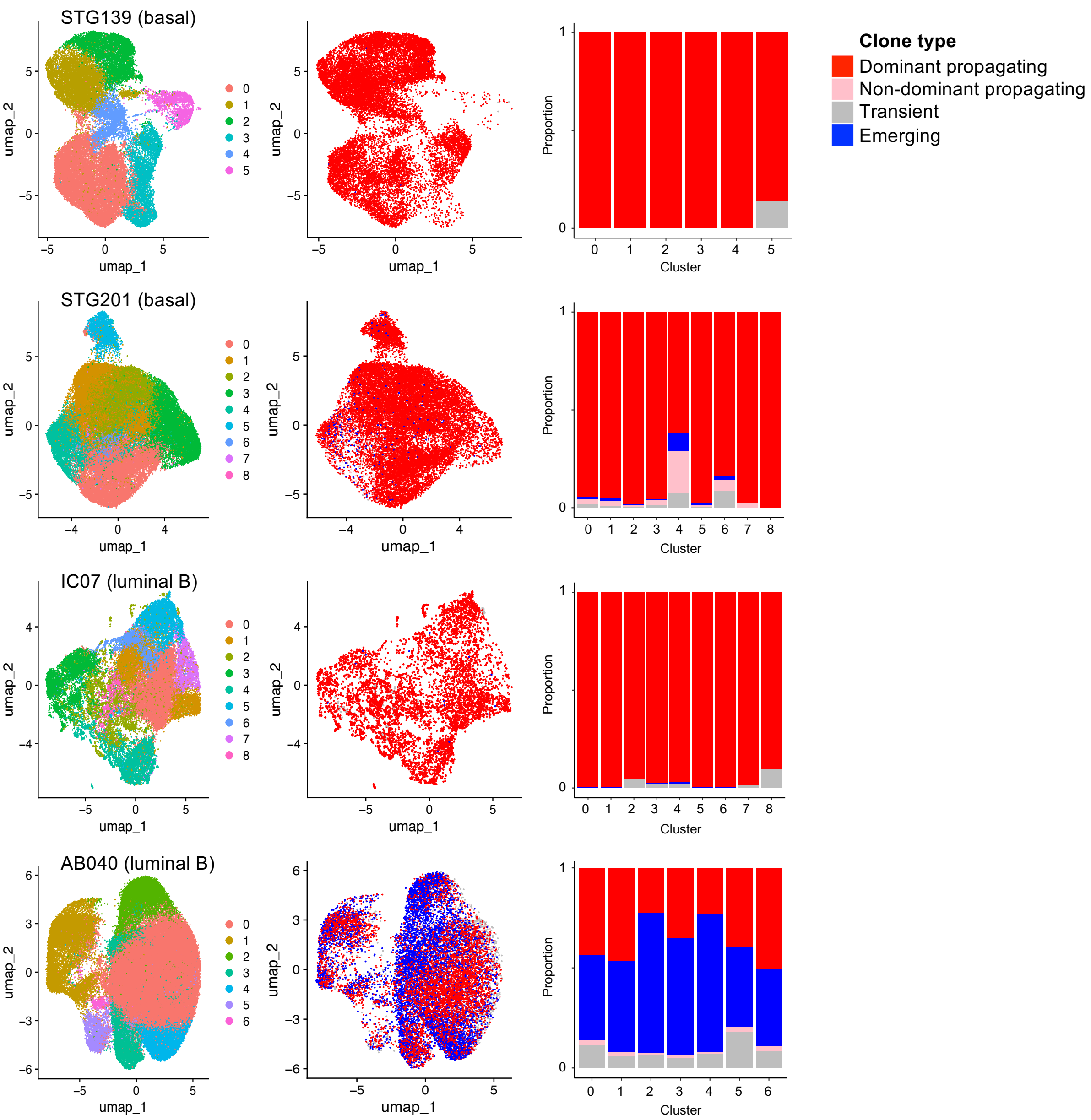

Figure S10. Distribution of clone types across Seurat clusters.
Data obtained from scRNAseq was analysed for each PDTX model using Seurat. UMAPs coloured by Seurat cluster and include all PDTX cells (barcoded and non-barcoded cells, left-most column) and coloured by clone type (barcoded cells only, middle column) are shown for each PDTX model. In the right-most column are bar plots showing the proportion of cells belonging to each clone type that contribute to each Seurat cluster.

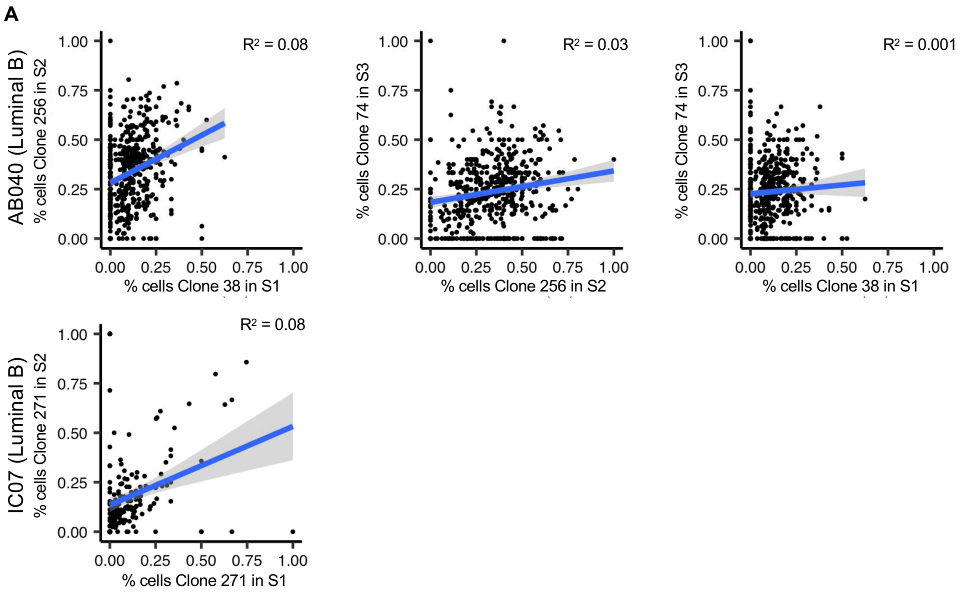

Figure S11. Cell state distribution of dominant clones

(A) Scatterplots showing the correlation of cell state proportions between secondary xenograft replicates for each dominant propagating clone: clone 38 in S1, clone 256 in S2 and clone 74 in S3 for AB040, and clone 271 in S1 and S2 for IC07. Each point represents a unique cell state, and the x and y axes represent the proportion contribution to that cell state by the clone out of total cells per cell state within each secondary xenograft replicate indicated. Blue lines show the linear correlations, with the shaded blue area indicating the standard error. Adjusted R^2 and p-values are also provided for each correlation.

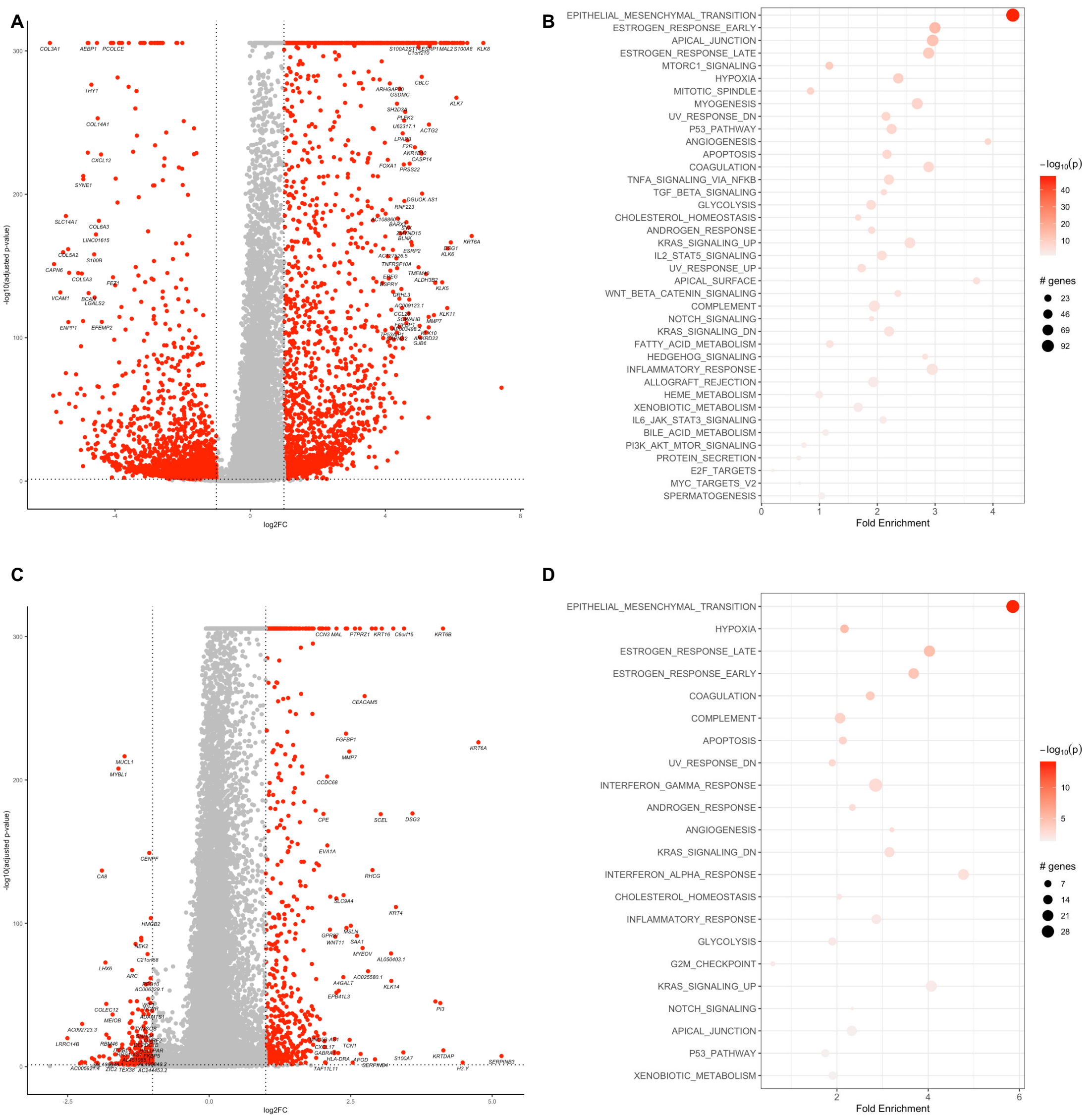

Figure S12. Differential gene expression analysis between dichotomous cell fractions from STG139 and STG201

(A) Volcano plot showing results from differential expression analysis comparing cells from Fraction 1 with cells from Fraction 2 in STG139. Statistically significant differentially expressed genes are indicated in red. Horizontal dotted line represents a significant adjusted p-value of -log10(0.05), and vertical dotted lines represent significant fold change of log2(2) or log2(0.5). A selection of the most significantly differentially expressed genes are labeled with their gene name. A positive log2-fold change indicates the gene is enriched in Fraction 1, and a negative log2-fold change indicates the gene is enriched in Fraction 2.

(B) Results of hallmark gene set enrichment analysis from genes that show statistically significant differential expression as identified in (A). The size of each point corresponds to the number of genes that show statistically significant differential expression in each gene set, and the intensity of the colour correspond to the significance shown as -log10(adjusted p-value).

(C) Volcano plot showing results from differential expression analysis comparing cells from Fraction 1 with cells from Fraction 2 in STG201. Statistically significant differentially expressed genes are indicated in red. Horizontal dotted line represents a significant adjusted p-value of -log10(0.05), and vertical dotted lines represent significant fold change of log2(2) or log2(0.5). A selection of the most significantly differentially expressed genes are labeled with their gene name. A positive log2-fold change indicates the gene is enriched in Fraction 1, and a negative log2-fold change indicates the gene is enriched in Fraction 2.

(D) Results of hallmark gene set enrichment analysis from genes that show statistically significant differential expression as identified in (C). Formatting is similar to (B).

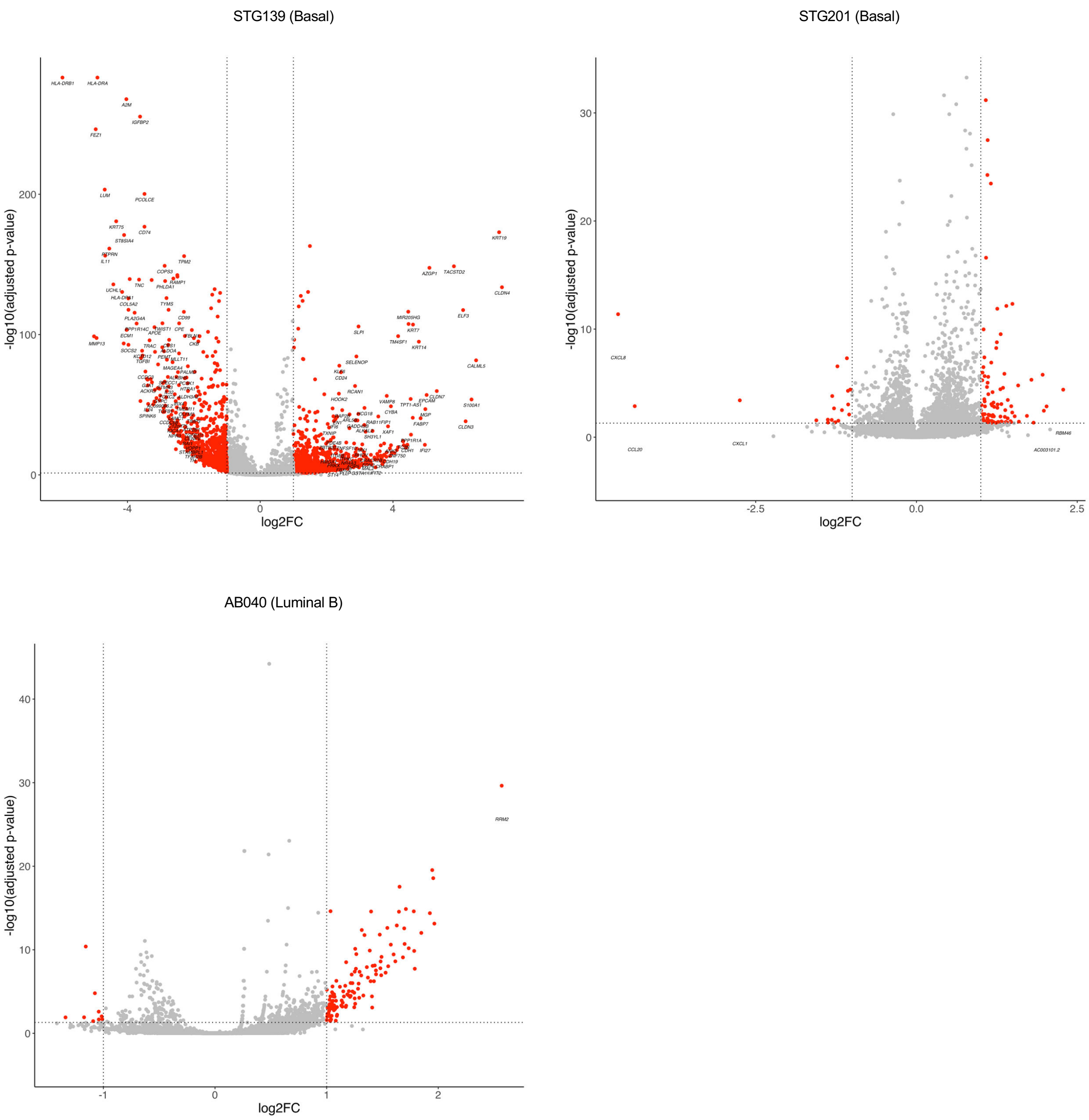

Figure S13. Volcano plots showing differential gene expression analysis between propagating and transient clones

Differential gene expression analysis was performed comparing cells from cell states unique only to propagating clones with cells from cell states unique only to transient clone in the primary xenograft only (i.e., before propagating activity is demonstrated in secondary xenografts). Statistically significant differentially expressed genes are indicated in red. Horizontal dotted line represents a significant adjusted p-value of -log10(0.05), and vertical dotted lines represent significant fold change of log2(2) or log2(0.5). A selection of the most significantly differentially expressed genes are labeled with their gene name. A positive log2-fold change indicates the gene is enriched in propagating clones, and a negative log2-fold change indicates the gene is enriched in transient clones.

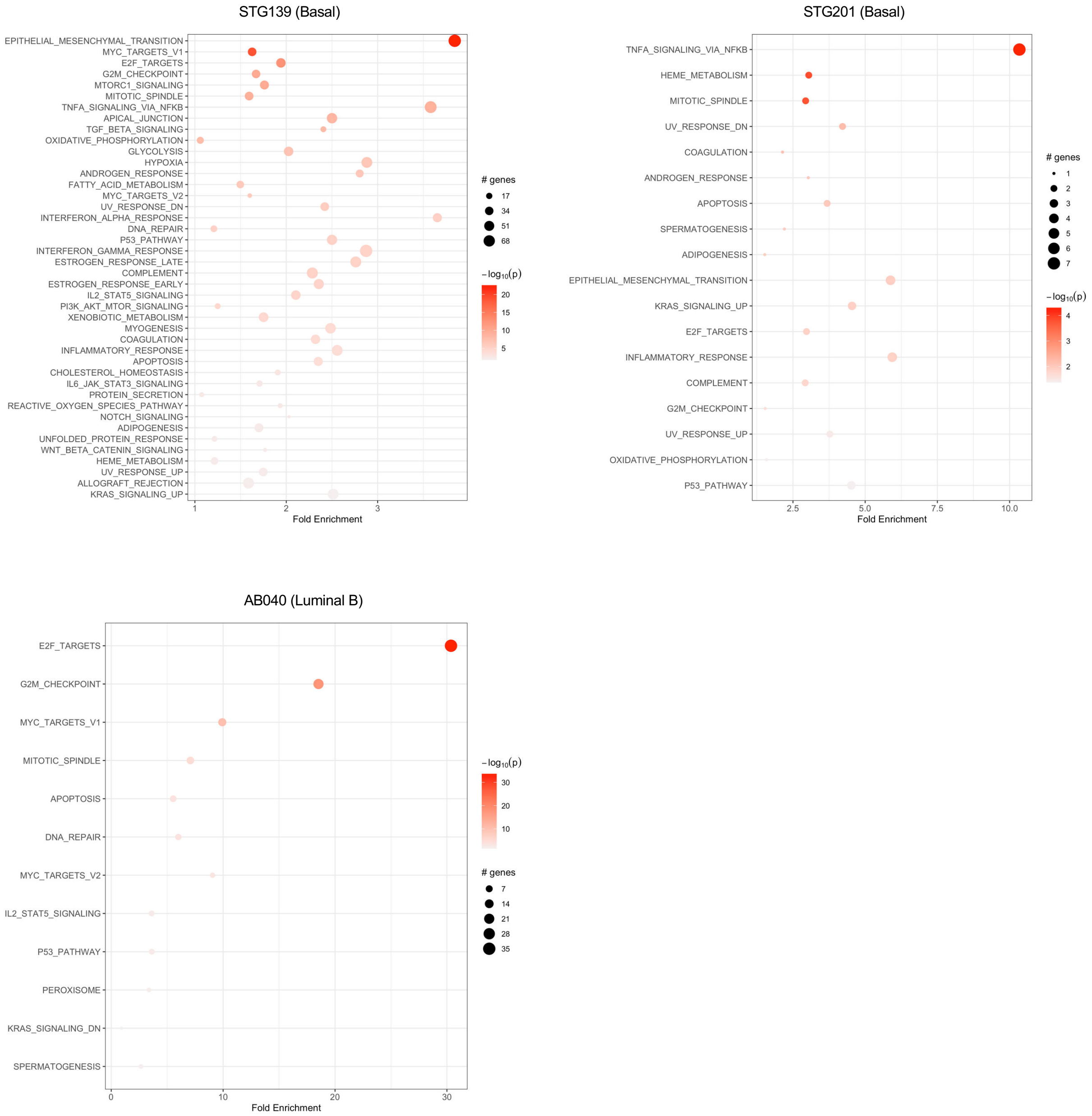

Figure S14. Hallmark gene set enrichment analysis between propagating and transient clones

Based on differentially expressed genes identified from analysis shown in Figure S8. The size of each point corresponds to the number of genes that show statistically significant differential expression in each gene set, and the intensity of the colour correspond to the significance shown as -log10(adjusted p-value).

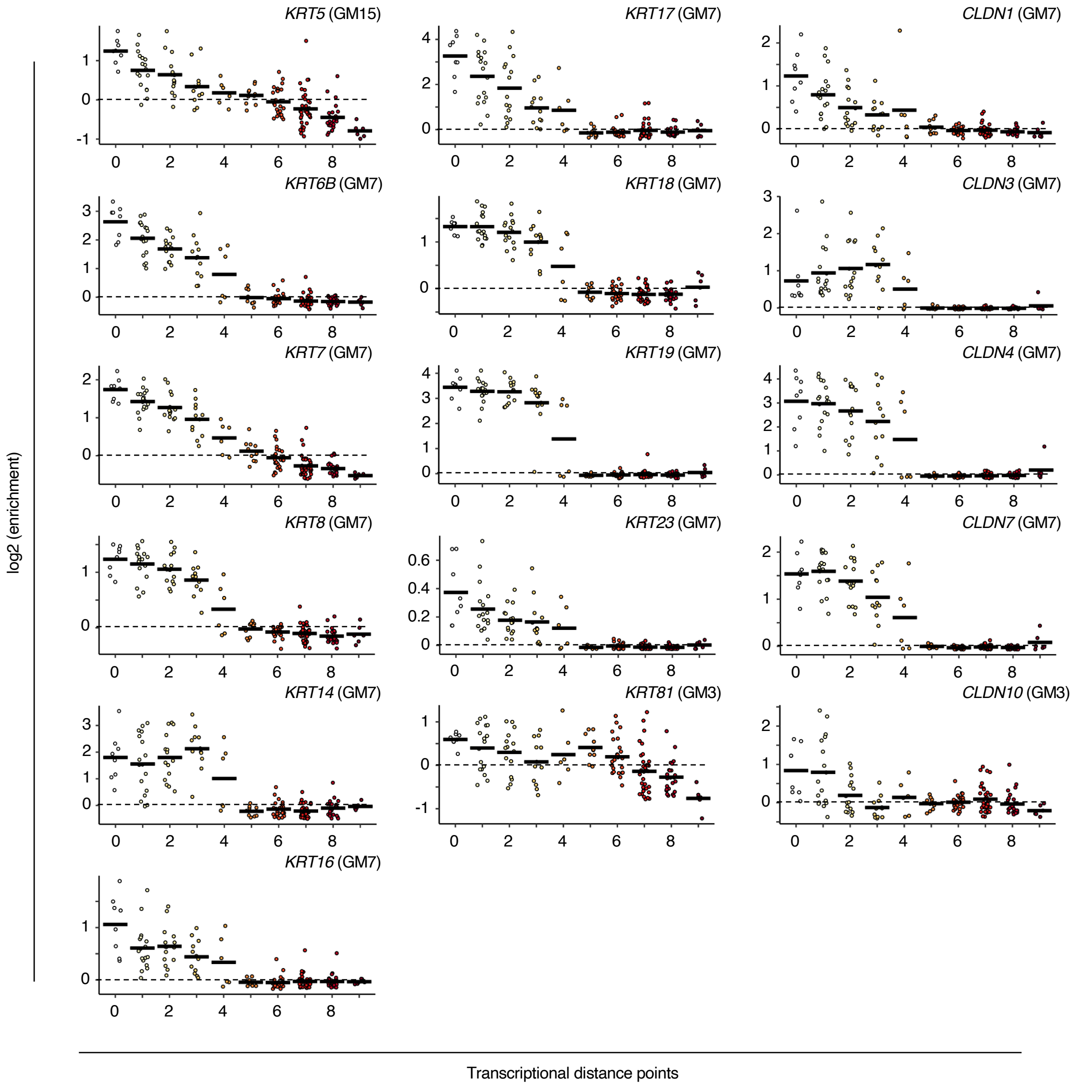

Figure S15. Individual gene enrichment plots for keratin and claudin genes over transcriptional distance for dominant propagating clone 1 from model STG139

The gene enrichment plots shown are for keratin and claudin genes pertaining to the gene modules indicated. Each plot shows the log2 enrichment of an individual gene. Each data point represents a single cell state grouped by distance points (along the x-axis), which are defined based on distance from the root cell state (distance 0). Horizontal black bars indicate the mean log2 enrichment. Horizontal dashed line is log2 of zero, indicating no positive or negative enrichment.

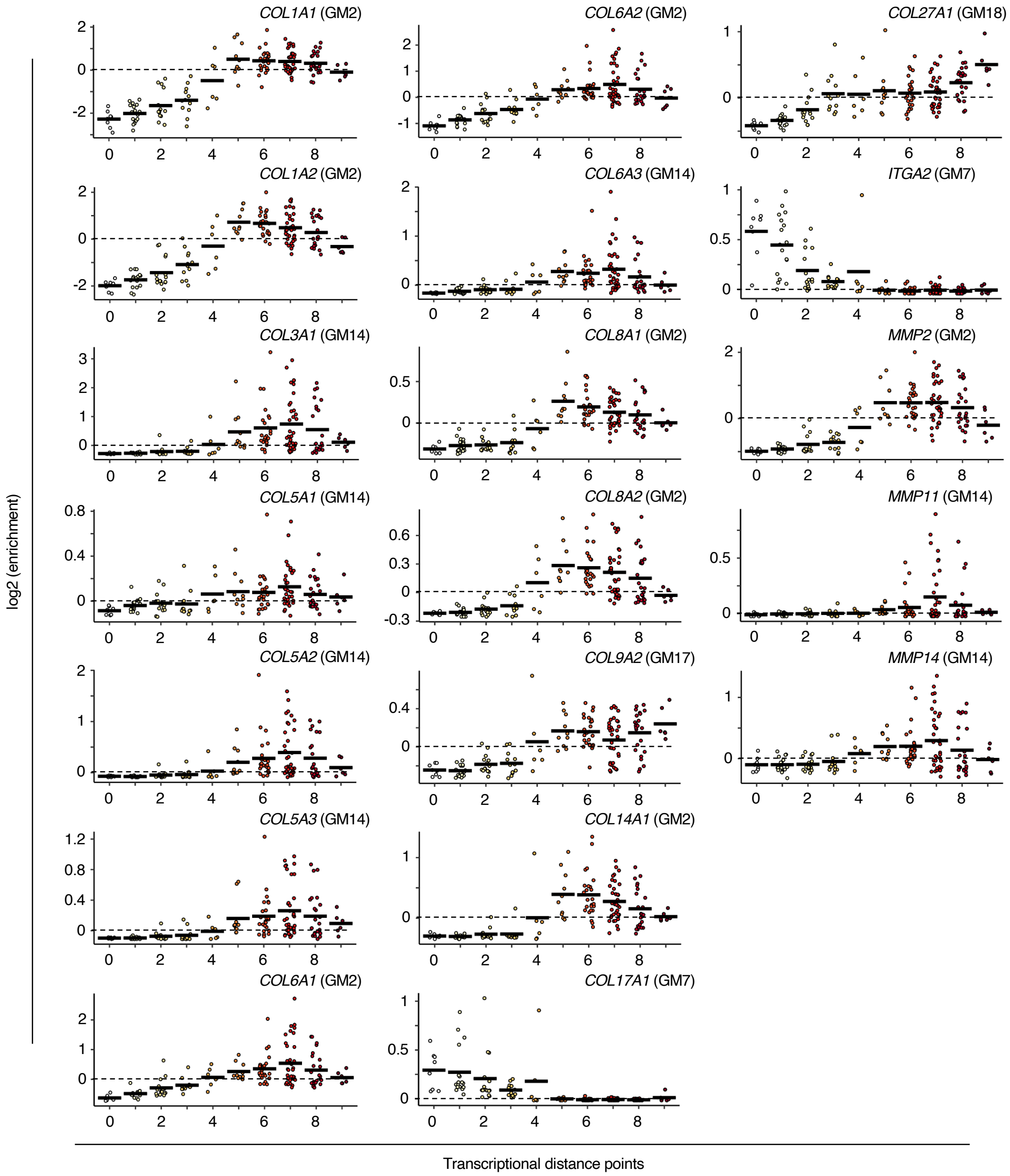

Figure S16. Individual gene enrichment plots for collagen, integrin and metalloproteinase genes over transcriptional distance for dominant propagating clone 1 from model STG139

The gene enrichment plots shown are for collagen, integrin, and metalloproteinase genes pertaining to the gene modules indicated. Each plot shows the log2 enrichment of an individual gene. Each data point represents a single cell state grouped by distance points (along the x-axis), which are defined based on distance from the root cell state (distance 0). Horizontal black bars indicate the mean log2 enrichment. Horizontal dashed line is log2 of zero, indicating no positive or negative enrichment.

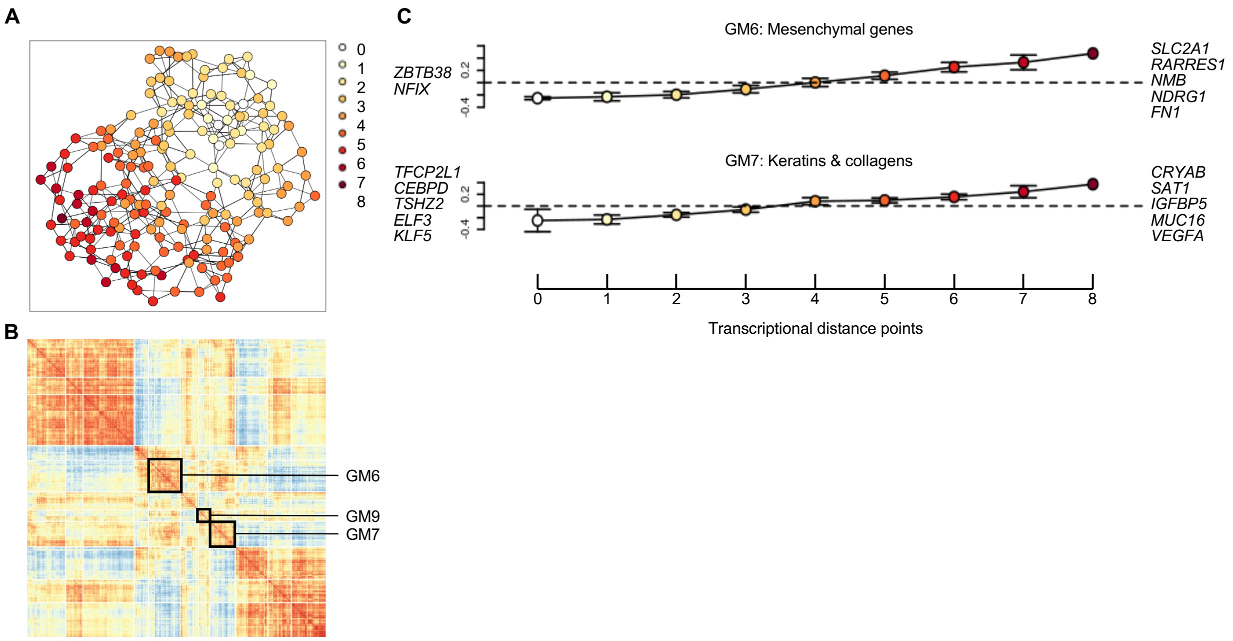

Figure S17. Transcriptional similarity analysis of dominant propagating clone 6 in STG201 reveals dynamic transcriptional plasticity

(A) Transcriptional similarity diagram of the transcriptional cell states in clone 6 of model STG201 represented in a 2-dimensional plot coloured by transcriptional proximity. The root of the diagram is designated as distance 0, with an additional 8 distance points identified corresponding to the distance of other cell states from the root cell state.

(B) Gene-gene correlation plot for STG201 where strong and highly variable expressed genes are clustered into 11 gene modules.

(C) Two plots show the fold-enrichment (y-axis) of the indicated gene modules over the cell states defined by distance point along the x-axis, with the colours of each point corresponding to the diagram in (A). Error bars are standard error of mean. Dotted horizontal line is centred around 0, indicating no enrichment of the gene module. Top 5 enriched transcription factors and genes (where they exist) within the gene module are indicated on the left and right of the plots, respectively.

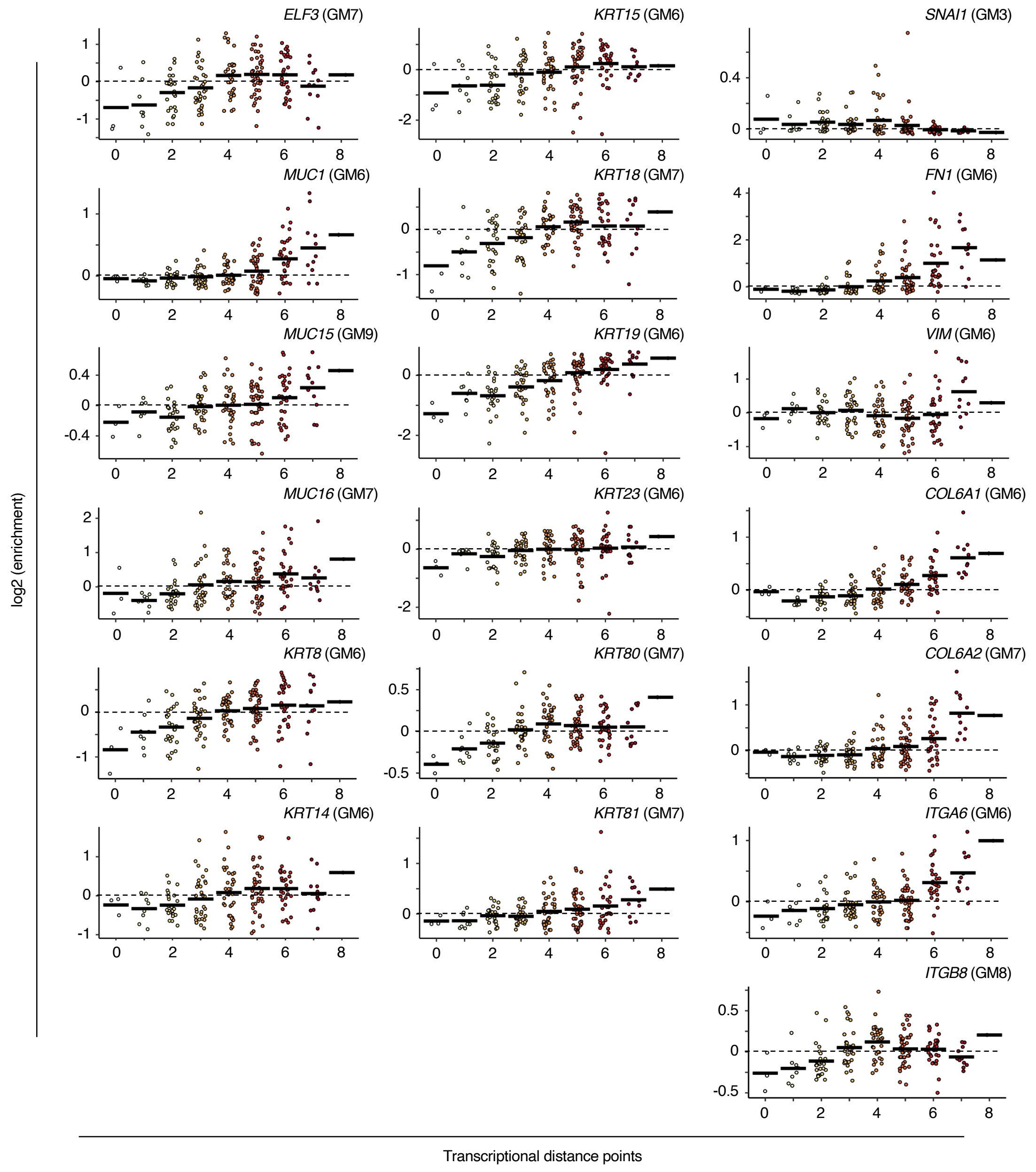

Figure S18. Individual gene enrichment plots over transcriptional distance for dominant propagating clone 6 from model STG201

Each plot shows the log2 enrichment of an individual gene. Each data point represents a single cell state grouped by distance points (along the x-axis), which are defined based on distance from the root cell state (distance 0). Horizontal black bars indicate the mean log2 enrichment. Horizontal dashed line is log2 of zero, indicating no positive or negative enrichment. The plots in the left and middle columns are epithelial, mucin and keratin genes, and right column are mesenchymal, collagen and integrin genes.

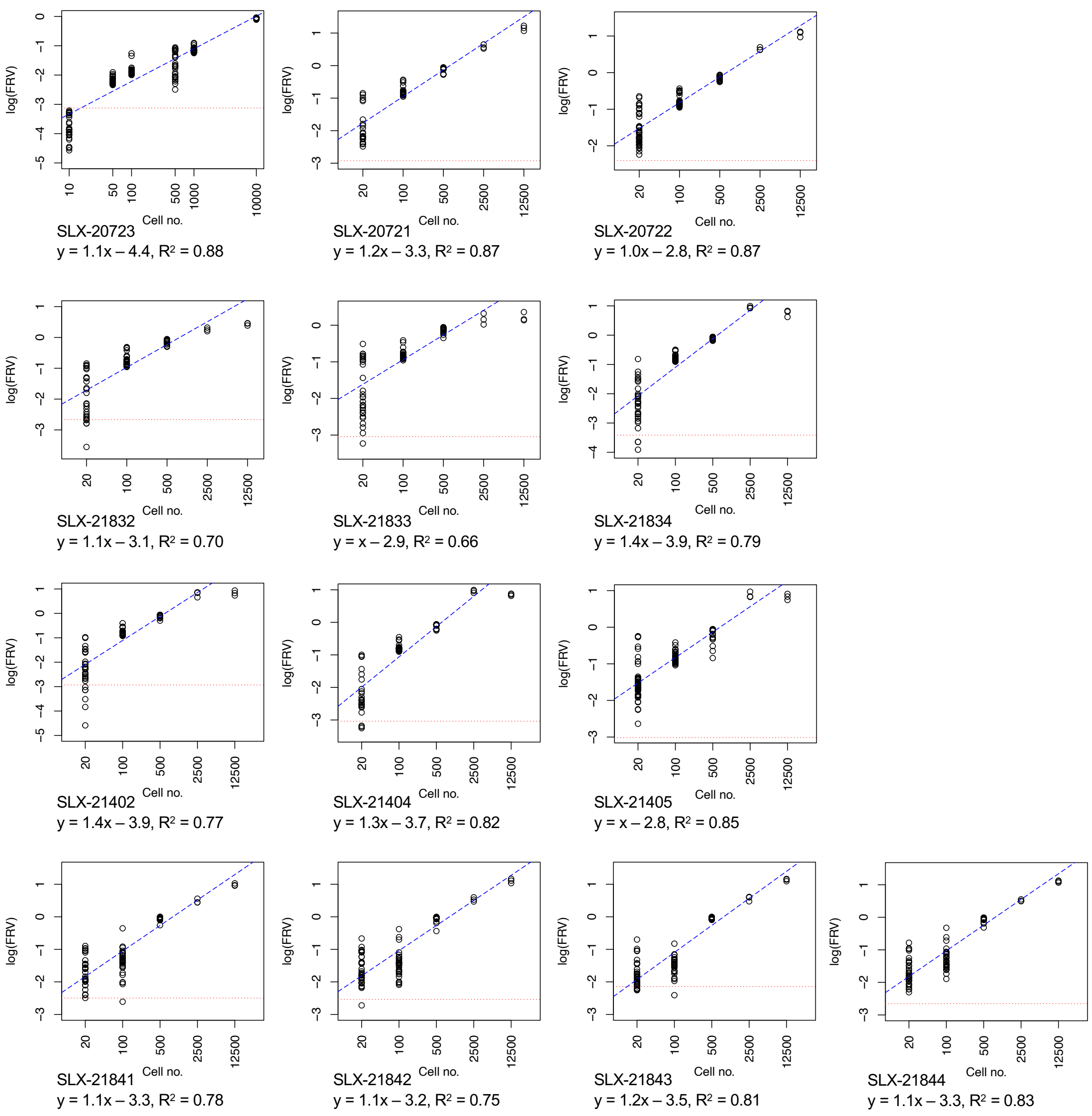

Figure S19 (Methods). Normalisation curves from multiplexed DNA amplicon sequencing to calculate absolute clone sizes from read count

For each run of multiplexed DNA amplicon sequencing, the log-log relationship between input cell dose per barcode clone and fractional read value is shown. This relationship allows for experimental clone size to be calculated based on normalized read count.

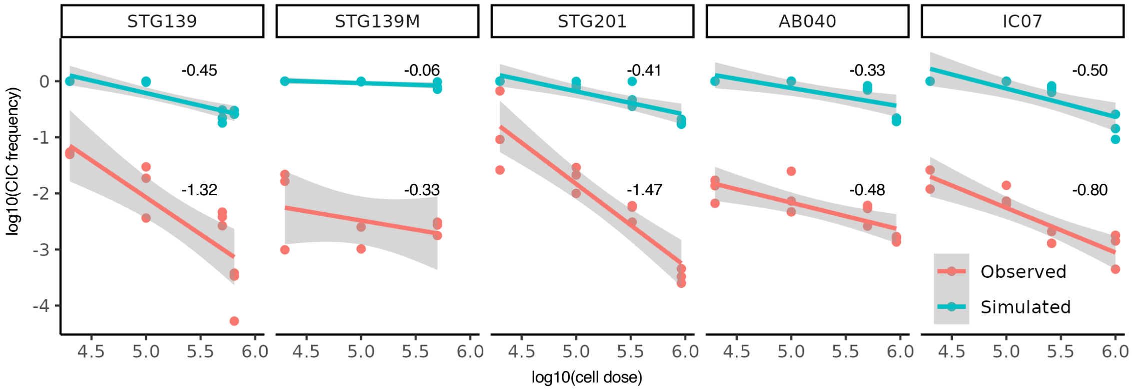

	STG139	STG139M	STG201	AB040	IC07
Corrected slope	-0.87	-0.27	-1.06	-0.15	-0.3

Figure S20 (Methods). In silico simulation of clone detection

Simulated versus observed negative log-log correlations between CIC frequency and cell dose are shown for each PDTX model. The slopes of these correlations are indicated beside the trendlines, with a table of the corrected slopes below, when the technical artefact of sampling proportionately fewer barcodes in tumours established with more barcoded cells (and thus represent a more diverse barcode pool) is removed (subtracted) from the experimentally observed slopes.

SUPPLEMENTARY TABLES

Table S2. Information on thresholds used in quality control and processing of the scRNAseq dataset. This includes the minimum number of UMI per cell, and the maximum fraction of mitochondrial UMI per cell.

Library name	Sample	Sequencing batch	UMI cutoff	Fraction mitochondrial UMI cutoff
LN_090822_3	STG139_P1	SLX-22050	4096	0.2
LN_160822_25	AB040_P1	SLX-22258	2048	0.2
LN_160822_26	IC07_S1	SLX-22258	4096	0.2
LN_160822_27	IC07_S2	SLX-22258	4096	0.2
LN_090822_1	STG139_S1	SLX-22050	2048	0.2
LN_090822_2	STG139_S2	SLX-22050	2048	0.2
LN_090822_10	STG201_S1	SLX-22050	4096	0.2
LN_090822_11	STG201_S2	SLX-22050	2048	0.2
LN_090822_12	STG201_S3	SLX-22050	4096	0.2
LN_100822_13	AB040_S1	SLX-22050	4096	0.2
LN_100822_14	AB040_S2	SLX-22050	2048	0.2
LN_100822_15	AB040_S3	SLX-22050	2048	0.2
STG201-X5_BC12LN	STG201_S1	SLX-22131	4096	0.2
STG201-X4	STG201_P1	SLX-22131	4096	0.2
STG139-X4	STG139_P2	SLX-22131	8192	0.2
IC07-X4	IC07_P1	SLX-22131	1024	0.35
NKI250-X2	NKI250_P1	SLX-22131	8192	0.2
AB040-X4	AB040_P2	SLX-21188	2048	0.2

Table S8. Sequencing depth and library diversity validation for barcode libraries BC1 and BC2.

Library	Sequencing ID	Replicate	Total reads passing quality filters	Total sequencing depth	Total unique barcodes
BC1 plasmid library	SLX-21845_UDP0273	1	4,850,774	30,071,925	1,056,379
	SLX-21845_UDP0274	2	4,683,313		
	SLX-21845_UDP0275	3	4,049,772		
	SLX-21401_UDP0017	4	1,159,223		
	SLX-21401_UDP0018	5	1,485,568		
	SLX-21401_UDP0019	6	956,469		
	SLX-20725_UDP0193	7	4,326,400		
	SLX-20725_UDP0194	8	3,527,657		
	SLX-20725_UDP0195	9	5,032,749		
BC2 plasmid library	SLX-22676_UDP0281	1	3,923,265	15,974,111	957,440
	SLX-22676_UDP0282	2	4,619,590		
	SLX-22676_UDP0283	3	4,085,378		
	SLX-21401_UDP0020	4	484,156		
	SLX-21401_UDP0021	5	934,859		
	SLX-21401_UDP0022	6	1,926,863		
BC1 transduced cells	SLX-20725_UDP0199	1	168,337	454,375	61,247
	SLX-20725_UDP0200	2	161,846		
	SLX-20725_UDP0201	3	124,192		
BC2 transduced cells	SLX-20725_UDP0202	1	196,686	511,746	93,905
	SLX-20725_UDP0203	2	167,170		
	SLX-20725_UDP0204	3	147,890		

Table S9. Transduction efficiency measurements for all PDTX experiments.

Model	Transduction efficiency (% GFP positive cells)
AB040	26
AB521T2	1.5
AB551	1
AB559	16
AB580	2.3
AB630	1.3
AB863T2	6.5
AB892T1	2.6
HCI001	3.8
HCI004	24.4
HCI009	4.7
HCI010	1.5
IC07	11 - 16
NKI127	13
NKI250	16
NKI336	7.1
STG139	18
STG139M	56
STG143	2.8
STG195	15.9
STG201	18
STG316	1.3
STG321	2.4
STG335	0.4
VHIO093	31
VHIO124	2.5

Table S10. Number of clones detected by scRNAseq for which more than one barcode sequence was detected.

This can represent either multiple barcode integrations, or cell doublets, the latter being a technical artefact from the scRNAseq platform.

Primary barcoded xenografts	Number of barcodes overlapping (subtracting those with matching pattern except 1)	Total barcode clones detected by amplicon sequencing	% clones with multiple barcodes (can be a consequence of multiple integration or cell doublets from scRNAseq)
AB040-X4_AN21-021169	28	1988	1.4
NKI250-X2_AN21-021169	21	998	2.1
IC07-X4_AN21-021162	29	654	4.4
STG139-X4_AN21-021161	0	337	0
STG201-X4_AN21-021165	30	616	4.9
TOTAL	108	4593	2.4

Table S11. Limits of clone detection calculated for all PDTX models.

PDTX model	Percentage of tumour sampled for barcode DNA amplicon sequencing		Threshold of clone detection (i.e. smallest clone size detectable with 95% confidence)	
	Lower range	Higher range	Lower range	Higher range
AB040	1	6	2000	333
AB521T2	1	2	2000	1000
AB551	5	17	400	118
AB559	4	10	500	200
AB580	2	100	1000	20
AB630	4	33	500	61
AB863T2	7	33	286	61
AB892T1	1	2	2000	1000
HCI001	2	3	1000	667
HCI004	3	20	667	100
HCI009	4	20	500	100
HCI010	3	4	667	500
IC07	1	13	2000	154
NKI127	4	20	500	100
NKI250	4	4	500	500
NKI336	10	20	200	100
STG139	3	7	667	286
STG139M	2	4	1000	500
STG143	33	50	61	40
STG195	2	4	1000	500
STG201	2	10	1000	200
STG316	3	4	667	500
STG321	1	4	2000	500
STG335	3	20	667	100
VHIO093	2	10	1000	200
VHIO124	2	4	1000	500

Table S12. Information on clone analysis from scRNAseq.

This includes the percentage of cells from scRNAseq with a detectable expressed GFP barcode sequence. This varied by sample, and in cases where the percentage GFP positive cells as analysed by flow cytometry was low, the proportion of GFP positive cells was enriched by cell sorting.

Sample	Xenograft passage	Number of cells passing QC threshold from scRNAseq	Number of cells detected with a barcode from scRNAseq	Number of unique barcodes	% Cells with a detectable barcode from scRNAseq	%GFP+ cells by flow cytometry	Enriched for GFP+ cells by cell sorting
STG139_P1	Primary	2206	67	4	3.0	4	No
STG139_P2	Primary	1317	980	23	74.4	26	Yes
AB040_P1	Primary	14961	1309	515	8.7	10	No
AB040_P2	Primary	5450	283	183	5.2	6	No
IC07_P1	Primary	6091	2573	11	42.2	2	Yes
STG201_P1	Primary	1063	314	54	29.5	19	Yes
NKI250_P1	Primary	164	96	16	58.5	12	Yes
**AVERAGE**	**Primary**	**31252**	**5622**	**806**	**18.0**		
STG139_S1	Secondary	11729	6379	1	54.4	6	Yes
STG139_S2	Secondary	12684	8768	1	69.1	30	Yes
AB040_S1	Secondary	15120	2276	20	15.1	20	No
AB040_S2	Secondary	17669	6713	15	38.0	42	No
AB040_S3	Secondary	14040	4031	23	28.7	32	No
IC07_S1	Secondary	12012	1134	1	9.4	4	No
IC07_S2	Secondary	11584	2117	4	18.3	10	No
STG201_S1	Secondary	1059	335	13	31.6	18	Yes
STG201_S1	Secondary	12829	5933	7	46.2	35	No
STG201_S2	Secondary	16317	7139	4	43.8	25	No
STG201_S3	Secondary	11080	4018	12	36.3	35	No
**AVERAGE**	**Secondary**	**136123**	**48843**	**101**	**35.9**		
**OVERALL**		**167375**	**54465**	**907**	**32.5**		

Table S15. Table of clustering parameters used for Seurat scRNAseq analysis.

Model	K-val	Resolution
AB040	20	0.2
IC07	30	0.3
STG139	15	0.2
STG201	20	0.3
